# IL-19 as a promising theranostic target to reprogram the glioblastoma immunosuppressive microenvironment

**DOI:** 10.1186/s12929-025-01126-w

**Published:** 2025-03-08

**Authors:** Gilbert Aaron Lee, Justin Bo-Kai Hsu, Yu-Wei Chang, Li-Chun Hsieh, Yi-Tien Li, Ying Chieh Wu, Cheng-Ying Chu, Yung-Hsiao Chiang, Wan-Yuo Guo, Chih-Chun Wu, Liang-Wei Chen, Hung-Wen Kao, Wan-Li Lin, Li‑Wen Tseng, Ting-Wei Weng, Duen-Pang Kuo, Sho-Jen Cheng, Yung-Chieh Chen, Shiu-Wen Huang, Hsing-Jien Kung, Cheng-Yu Chen

**Affiliations:** 1 Department of Medical Research, Taipei Medical University Hospital, Taipei, Taiwan; 2 Department of Microbiology and Immunology, School of Medicine, College of Medicine, Taipei Medical University, Taipei, Taiwan; 3 Child Development Research Center, Taipei Medical University Hospital, No. 250, Wu Hsing Street, Taipei, 110 Taiwan; 4 TMU Research Center for Digestive Medicine, Taipei Medical University, Taipei, Taiwan; 5 Department of Computer Science and Engineering, Yuan Ze University, Taoyuan, Taiwan; 6 Department of Radiology, School of Medicine, College of Medicine, Taipei Medical University, No. 250, Wu Hsing Street, Taipei, 110 Taiwan; 7 Department of Medical Imaging, Taipei Medical University Hospital, Taipei, Taiwan; 8 Translational Imaging Research Center, Taipei Medical University Hospital, Taipei, Taiwan; 9 Neuroscience Research Center, Taipei Medical University, Taipei, Taiwan; 10 Ph.D. Program in Medical Neuroscience, College of Medical Science and Technology, Taipei Medical University, Taipei, 11031 Taiwan; 11 CRISPR Gene Targeting Core, Taipei Medical University, Taipei 110, Taiwan; 12 TMU Research Center of Cancer Translational Medicine, Taipei Medical University, Taipei 110, Taiwan; 13 Department of Surgery, College of Medicine, Taipei Medical University, Taipei, Taiwan; 14 Department of Radiology, Taipei Veterans General Hospital, Taipei, Taiwan; 15 Radiological Diagnosis Department, Tri-Service General Hospital, Taipei, Taiwan; 16 Department of Nuclear Medicine, Tri-Service General Hospital, National Defense Medical Center, Taipei, Taiwan; 17 Department of Pharmacology, School of Medicine, College of Medicine, Taipei Medical University, Taipei, Taiwan; 18 Graduate Institute of Medical Sciences, College of Medicine, Taipei Medical University, Taipei, Taiwan; 19 Research Center of Cancer Translational Medicine, Taipei Medical University, Taipei, Taiwan

**Keywords:** IL-19, Glioblastoma, Temozolomide

## Abstract

**Background:**

Glioblastoma multiforme (GBM) is an aggressive brain tumor with chemoresistant, immunosuppressive, and invasive properties. Despite standard therapies, including surgery, radiotherapy, and temozolomide (TMZ) chemotherapy, tumors inevitably recur in the peritumoral region. Targeting GBM-mediated immunosuppressive and invasive properties is a promising strategy to improve clinical outcomes.

**Methods:**

We utilized clinical and genomic data from the Taiwan GBM cohort and The Cancer Genome Atlas (TCGA) to analyze RNA sequencing data from patient tumor samples, determining the association of *interleukin-19* (*Il-19)* expression with survival and immunosuppressive activity. Gene set enrichment analysis (GSEA) was performed to assess the relationship between the enrichment levels of immune subsets and *Il-19* expression level, and Ingenuity Pathway Analysis (IPA) was used to predict immune responses. Cytokine array and single-cell RNA sequencing were used to examine the effects of IL-19 blockade on tumor immune microenvironment, including tumor-infiltrating leukocyte profiles, differentiation and immunosuppressive genes expression in tumor associated macrophages (TAM). CRISPR *Il-19*^−/−^ cell lines and *Il-19*^−/−^ mice were used to examine the role of IL-19 in tumor invasion and M2-like macrophage-mediated immunosuppression. Additionally, we developed novel cholesterol-polyethylene glycol-superparamagnetic iron oxide-IL-19 antibody nanoparticles (CHOL-PEG-SPIO-IL-19), characterized them using dynamic light scattering and transmission electron microscopy, Fourier-Transform Infrared spectroscopy, prussian blue assay, and conducted in vivo magnetic resonance imaging (MRI) in a human glioblastoma stem cell-derived GBM animal model.

**Result:**

Genomic screening and IPA analysis identified IL-19 as a predicted immunosuppressive cytokine in the peritumoral region, associated with poor survival in patients with GBM. Blocking IL-19 significantly inhibited tumor progression of both TMZ-sensitive (TMZ-S) and TMZ-resistant (TMZ-R) GBM-bearing mice, and modulated the immune response within the GBM microenvironment. Single-cell transcriptome analysis reveal that IL-19 antibody treatment led to a marked increase in dendritic cells and monocyte/macrophage subsets associated with interferon-gamma signaling pathways. IL-19 blockade promoted T cell activation and reprogrammed tumor-associated macrophages toward weakened pro-tumoral phenotypes with reduced Arginase 1 expression. *Il19*^−/−^ M2-like bone marrow-derived macrophages with lower Arginase 1 level lost their ability to suppress CD8 T cell activation. These findings indicated that IL-19 suppression limits TAM-mediated immune suppression. Molecular studies revealed that IL-19 promotes TMZ-resistant GBM cell migration and invasion through a novel IL-19/WISP1 signaling pathway. For clinical translation, we developed a novel CHOL-PEG-SPIO-IL-19 nanoparticles to target IL-19 expression in glioblastoma tissue. MRI imaging demonstrated enhanced targeting efficiency in brain tumors, with in vivo studies showing prominent hypointense areas in T2*-weighted MRI scans of tumor-bearing mice injected with CHOL-PEG-SPIO-IL-19, highlighting nanoparticle presence in IL-19-expressing regions. Prussian blue staining further confirmed the localization of these nanoparticles in tumor tissues, verifying their potential as a diagnostic tool for detecting IL-19 expression in glioblastoma. This system offers a theranostic approach, integrating diagnostic imaging and targeted therapy for IL-19-expressing GBM.

**Conclusion:**

IL-19 is a promising theranostic target for reversing immunosuppression and restricting the invasive activity of chemoresistant GBM cells.

**Graphical Abstract:**

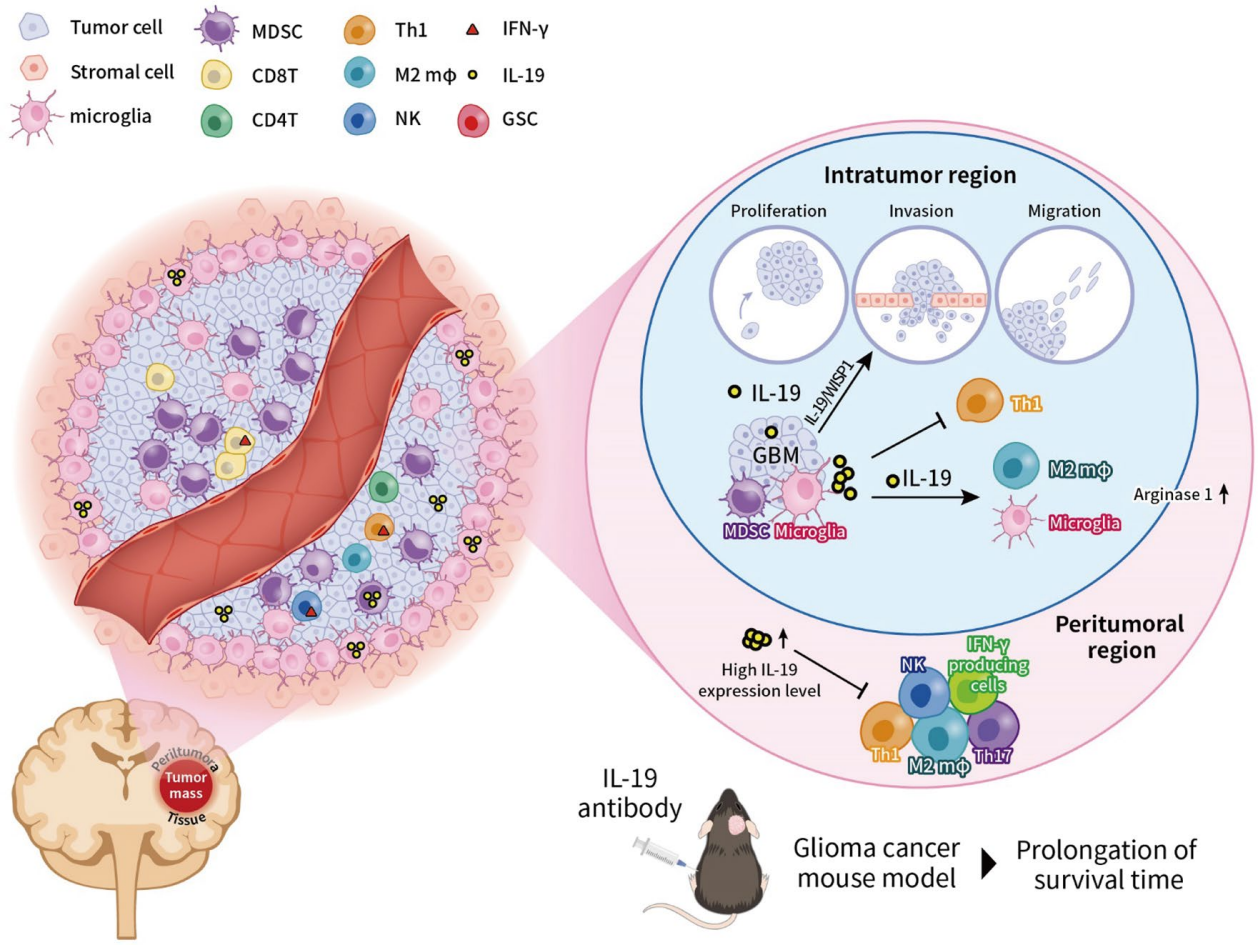

**Supplementary Information:**

The online version contains supplementary material available at 10.1186/s12929-025-01126-w.

## Introduction

Glioblastoma multiforme (GBM) is the most common malignant brain tumor with a recurrence rate of over 90% [51]. Even with complete surgical resection followed by chemoradiotherapy, recurrence occurs in almost all affected patients, primarily at the margin of the resected tumor bed, which is referred to as the peritumoral region [55, 57]. The peritumoral region contains tumor and stromal cells that promote the growth and invasion of GBM [37]. Tumor cell infiltration into the peritumoral region may promote recurrence [47, 68]. Given the heterogeneity of GBM, large-scale studies are required to analyze the genetic and molecular characteristics of cells in the peritumoral region where new molecular targets can be identified to prevent recurrence.

Most recurrent GBM is characterized by an immunosuppressive tumor microenvironment [58, 64]. Myeloid-derived suppressor cells (MDSCs) and tumor-associated macrophages (TAMs) are immunosuppressive subsets that inhibit the function of T cells and cytotoxic natural killer (NK) cells within tumors [10, 46, 53]. TAMs secrete arginase 1 (Arg1), which depletes arginine and limits T cell activation and antitumor responses [18]. Blocking intratumoral Arg1-mediated T cell exhaustion may help reverse the immunosuppressive environment in GBM. Although immunotherapy holds potential for improving clinical outcomes and prolonging survival in various cancers, its efficacy in GBM remains limited, potentially due to tumor heterogeneity [9, 48]. Intratumoral heterogeneity across different regions of individual tumors means that small biopsy specimens may not accurately represent the molecular profile of the entire tumor [4, 27]. Moreover, various analytic strategies in immunohistochemistry and inconsistent cut-off values for target gene expression can lead to false classification, depending on the area of the tumor sampled [19, 20]. Therefore, a standardized approach for quantifying target expression across the whole tumor is necessary to improve consistency and accuracy in GBM diagnosis and treatment [56]. Additionally, intertumoral heterogeneity between different patients with GBM raises the challenge of accurately stratifying the immune response in each individual. This is crucial for achieving better efficacy in targeted immunotherapy.

Superparamagnetic iron oxide (SPIO) nanoparticles represent a new type of magnetic resonance imaging (MRI) contrast agent for biomedical imaging applications [16, 54, 67]. Studies have shown that nanoparticles smaller than 100–200 nm can pass through the blood–brain barrier (BBB) via the enhanced permeability and retention (EPR) effect, serving as a passive tumor-targeting system [66]. However, the efficacy of EPR in delivering drugs to tumor sites is suboptimal due to macrophage clearance [71]. To address this issue, various modifications have been explored, leading to the development of a dual-modified drug delivery system based on polyethylene glycol (PEG) and cholesterol [25]. The classical bonds between hydrophobic and hydrophilic moieties, such as the ether linkage (CHOL-PEG), are noncleavable, resulting in a highly stable hydrated layer surrounding the SPIO nanoparticles. This structure increases the nanoparticles' affinity for the BBB and prolongs circulation time [60]. This material may enhance the targeting efficiency of SPIO nanoparticles by improving their affinity for the BBB and enhancing the EPR effect.

Given that immune genes are associated with the survival of patients with GBM [62], we aim to identify the most critical immune-related gene that are associated with patients’ survival by genomic screening approach. In this study, we identified a novel therapeutic target, Interleukin-19 (IL-19), which is a poor prognosis factor in patients with GBM. IL-19 functions through a receptor complex composed of interleukin-20 receptor subunit alpha (IL-20RA) and interleukin-20 receptor subunit beta (IL-20RB) and its downstream signaling is mediated by STAT3 phosphorylation [15]. *IL-19* expression is positively correlated with tumor metastasis and clinical outcomes in patients with breast cancer and esophageal squamous cell carcinoma [23, 24]. IL-19 promotes breast cancer cell invasion and induces the phosphorylation of ERK, p38, and AKT [23, 32]. It is primarily produced by activated macrophages and microglia [3, 22]. IL-19 exerts anti-inflammatory effects on macrophages by inhibiting inflammatory cytokine production, downregulating antigen-presenting capacity, and promoting M2 phenotype polarization [13, 21, 36]. Due to its immunosuppressive activity, IL-19 could serve as an immunoregulatory cytokine by dampening antigen-presenting capacity and regulating M2 macrophage polarization in the tumor microenvironment. However, the role of IL-19 in the GBM tumor microenvironment remains unknown.

In this study, we demonstrated the immunosuppressive function of IL-19 in the GBM tumor microenvironment through single-cell RNA sequencing (scRNA-seq) analysis. Blocking IL-19 boosted CD8 T cell activation by weakening TAM-mediated immunosuppressive activity. IL-19 promotes TMZ-resistant GBM invasion via a novel IL-19 / AKT / WNT1-inducible signaling pathway protein 1 (WISP1) axis. Furthermore, a novel IL-19-targeting nanoparticle (IL-19 antibody conjugated with CHOL-PEG-SPIO-modified nanoparticles, CHOL-PEG-SPIO-IL-19) was developed to detect IL-19 expression levels in human GBM tumors. These IL-19-targeting nanoparticles can be used to select patients with GBM who are likely to respond to IL-19 antibody therapy. Thus, IL-19 is a promising theranostic target, acting as a double-edged sword to abrogate TAM-mediated immunosuppressive effects and tumor cell invasiveness.

## Materials and methods

### Human study cohort

This study was comprised of three cohorts. First, this study enrolled a local Taiwanese cohort consisting of 28 patients with GBM with complete MRI sets and microarray genomic data obtained from intratumoral and peritumoral tissues through stereotactic sampling. The second cohort was obtained from TCGA database, which included the genomic and survival data of 225 patients with GBM. The third cohort was obtained from CGGA database (mRNA_array_301). All GBM had wild-type isocitrate dehydrogenase 1 (IDH-1). This study was approved by the Institutional Review Board of Taipei Medical University (approval no. N201603086).

### Immune subset enrichment level estimation

GBM RNA-seq datasets from TCGA level 1 data were processed and analyzed using HISAT2 or StringTie [69]. The array platform employed for the GBM cohort was Agilent 244 K custom gene expression (G4502A-07–2; Agilent, CA, USA). The gene intensity was normalized using locally weighted scatterplot smoothing. The enrichment levels of various immune subsets, namely T_H_1, T_H_2, T_H_17, cytotoxic T lymphocytes (CTLs), and MDSCs, were evaluated through gene set enrichment analysis based on TCGA Level 1 data. A metagene list is presented in Supplementary Table S1.

### Genomic screening of immune-related genes associated with patient survival and survival curve analysis

A total of 8,901 immune-related genes were selected from ImmPort, InnateDB, GO, and the Panther database. Putative survival-associated immune-related genes were ranked and filtered using stringent criteria (hazard ratio [HR] > 1; Wald test, *p* < 0.01). The association between *IL-19* expression level and patient prognosis was assessed using the Cox proportional hazards model, implemented through the coxph function in the R package survival. The HR derived from this model quantified the effect of a one-unit increase in *IL-19* expression on the risk of an event occurring. To further examine the impact of *IL-19* expression, the cutp function from the R package survMisc was used to identify the optimal cut-off point for stratifying patients into high- and low-expression groups. Using this cut-off value, Kaplan–Meier survival curves were generated to visualize the survival differences between the two groups. The statistical significance of these differences was evaluated using the log-rank test.

### RNA microarray and ingenuity pathway analysis of peritumoral and intratumoral GBM tumor tissue

Tissue samples were collected from the intratumoral solid T1 contrast-enhanced tissue and the peritumoral region (tumor-free resection margins of 1–2 cm), which was defined using multimodal magnetic resonance (MR) images (T1, T1 contrast-enhanced tissue (T1 + C), T2 Fluid Attenuated Inversion Recovery, and apparent diffusion coefficient maps); the samples were then obtained using MR-guided stereotaxic surgery. All RNA was extracted from the tumor tissue and hybridized to an Agilent SurePrint Microarray (Agilent Technologies, Design ID: 039494), and the signals were analyzed using feature extraction software (v10.7.3.1; Agilent). An Ingenuity Pathway Analysis (IPA) system (version 42012434, Ingenuity Systems; Qiagen, China) was used for the bioinformatics analysis.

### Human tumor tissue immunofluorescence staining

The tumor tissues were fixed with 4% paraformaldehyde for approximately 3 days, then were embedded in paraffin and cut into 5-µm-thick sections, which were deparaffinized and rehydrated using a graded ethanol series. The sections were then exposed to an antigen retrieval process, and sections were stained using IL-19 antibody (R&D systems, #AF1035, 100X dilution), CD206 antibody (Cell signaling, E2L9N, 800X dilution), Iba1 antibody (Cell signaling, E4O4W, 200X dilution). The secondary antibodies were Alexa Fluor 488 AffiniPure donkey anti-rabbit IgG (Jackson ImmunoResearch Laboratories, 500X dilution), and Cy3 donkey anti-goat antibody (Abcam, #ab150, 600X dilution). The sections were observed by a light microscope (Olympus/Bx43).

### GBM cell lines and glioblastoma stem cells

Human GBM cell line, including U87, GBM8401, GBM8901, and DBTRG-05MG cell lines were purchased from the Bioresource Collection and Research Center (Taiwan). U118 and LN18 cells were purchased from (American Type Culture Collection, ATCC). Mouse GBM cell line, GL261 cell line, was purchased from the German Collection of Microorganisms and Cell Cultures (Leibniz Institute, DSMZ, Germany). Human glioblastoma stem cells (GSC) were purchased from Celprogen (USA). GL261 cells were transfected with the plasmid vector PGL4.51 (luc2/CMV/Neo). GL261 TMZ-resistant subline (GL261/TMZ-R) cells were established according to our previous study [35]. The DBTRG TMZ-resistant subline (DBTRG/TMZ-R) was generated by long-term incubation with 50 μM TMZ. TMZ-resistance activity was confirmed by a colony formation assay (Supplementary Fig. 1).

### Glioma-bearing animal model

C57BL/6JNarl male mice (National Laboratory Animal Center, Taiwan) were used to establish a GBM-bearing animal model. NBSGW mice (NOD, B6.SCID Il2rγ^−/−^ Kit^W41/W41^ mice, Jackson Lab) [45] were used for human GBM-bearing animal model. The tumor cell inoculation assay was performed as previously described in another study [30]. Briefly, 100,000 GL261 or 150,000 GL261/TMZ-R cells were slowly injected into the right brain of C57BL/6JNarl male mice. IL-19 antibody (R&D Systems) or an isotype control antibody was intravenously administered on days 12, 15, 19, and 22 (15 μg/mouse). Tumor volume was analyzed using a Spectrum In Vivo Imaging System (IVIS Lumina III XRMS, PerkinElmer), and the animals were monitored daily to ensure humane endpoints for animal survival experiments. This animal study was approved by the Institutional Animal Care and Use Committee of Taipei Medical University (LAC 2018-0478) and conducted in accordance with the Animal Research: Reporting of In Vivo Experiments (ARRIVE) guidelines and the Guide for the Care and Use of Laboratory Animals of the National Institutes of Health.

### Tumor cytokine expression profile

The cytokine profiles of the tumor homogenates were analyzed using the Proteome Profiler Mouse XL Cytokine Array Kit (ARY028; R&D Systems, USA) in accordance with the manufacturer’s instructions. We used 200 μg of the tissue lysates of tumor homogenates to determine cytokine expression profiles. All data were acquired using the ChemiDoc Touch Imaging System (Bio-Rad, USA) and signals were quantified using ImageJ software.

### Tumor-infiltrating leukocyte scRNA-seq analysis

ScRNA-seq analysis was performed on day 25 after tumor inoculation to isolate tumor-infiltrating leukocytes from tumor-bearing mice. Mice were anesthetized with zoletil and xylazine and transcardially perfused with cold phosphate-buffered saline (PBS). Tumor tissues were dissociated into single-cell suspensions using a tumor dissociation kit (Miltenyi Biotec) in combination with a gentleMACS Octo Dissociator with Heaters (Miltenyi Biotec). The BD Rhapsody Express Single-Cell Analysis System was used for all the targeted transcriptomic experiments. We first isolated CD45^+^ live tumor-infiltrating leukocytes and then labeled specific markers (AbSeq Oligo anti-mouse CD3, CD4, CD8, NKp46, CD11b, and CD11c) using a BD Single-Cell Multiplexing Kit (BD Biosciences, #633781, USA) and BD AbSeq Ab-Oligos reagents according to the manufacturer’s protocol. The final libraries were sequenced using the HiSeq 2500 sequencing system (Illumina, USA), which generated paired-end reads of 150 base pairs. The final mean read depths for the two experiments were as follows: Experiment 1 had 8467 reads/cell for the AbSeq library (saturation: 96.5%) and 12,204 reads/cell for the mRNA library (saturation: 67.4%). Experiment 2 had 9950 reads/cell for the AbSeq library (saturation: 97.1%) and 26,728 reads/cell for the mRNA library (saturation: 76.6%). Whole-transcriptome FASTQ files were processed using the standard Rhapsody WTA Analysis Pipeline (version 1.9.1; BD Biosciences) on the Seven Bridges platform, and immune subset clustering was implemented using the Seurat plugin (version 3.8.1) in SEQGEQ software (BD Bioscience). Raw scRNA-seq data were deposited in PRJNA1036784. The DEGs of immune subsets were showed in supplementary file 1.

### Generation of *Il-19* knockout cell line through CRISPR/Cas9-mediated genome editing

*Il-19* knockout (IL-19KO) GL261/TMZ-R cells were generated using CRISPR/Cas9. Two single-guided RNAs (sgRNAs) targeting the second and fourth coding exons of *Il-19* were separately cloned into pAll-Cas9. The pPpuro plasmid was obtained from the National RNAi Core Facility (Academia Sinica, Taipei, Taiwan). The sequences of the targeting sites were as follows: 5′- AGAGAATCAACGTCATGCCCAGG -3′ for sgRNA#1, and 5′- GTAGAATGTCAGCAGGTTGTTGG -3′ for sgRNA#2. A control sgRNA targeting EGFP was cloned and used in this study. The two sgRNA plasmids used to generate the *Il-19* gene KO GL261/TMZ-R cells were transfected into GL261 cells using the Lonza 4D Nucleofector X Unit (Lonza). Two days post-transfection, the cells were cultured with 2 μg/mL puromycin for 1 week. Viable cells were subjected to limiting dilution in a 96-well plate to isolate single-cell clones. IL-19KO cells were verified by western blot analysis and DNA sequencing of genomic regions.

### Generation of *Il-19*^*−/−*^ mice

*Il-19*^−/−^ mice were generated using CRISPR/Cas9-mediated fragment deletion. Two crRNAs (IDT) targeting introns 1 and 4 were used to generate the *Il-19* knockout allele. The crRNA sequences used were 5′-TAGGGATCCTGAAAATGTAG and 5′-GGACTTGGTCCCATGCTAAG, respectively. Super-ovulated 3–4-week-old C57BL/6JNarl female mice were mated with male mice, and one-cell stage zygotes were collected the following day. A mixture of crRNA-tracrRNA and Cas9 recombinant protein (IDT#1,081,061) at concentrations of 0.15 μM, 0.15 μM, and 1.8 μM, respectively, was injected into the cytoplasm of the zygotes. Injected zygotes were transferred into the oviducts of 0.5-dpc pseudo-pregnant ICR female mice (BioLASCO Taiwan). To confirm the knockout allele, three primers (Il19-Fw: 5′-CTACAGTCTTAGGAGATGTCTGATTTCTGT; Il19-Rv: 5′-GTCTACCCAAACATCACACAGC; and Il19 int2-Rv: 5′-CCAAGCCACTGAAATTCTGCCC) were used to differentiate the knockout and wild-type (wt) alleles. The knockout allele and wt allele resulted in amplicons of 278 bp and 531 bp, respectively. Two knockout mouse lines (*Il-19*^−/−^-K1 and *Il-19*^−/−^-K2) were generated.

### DNA constructs and lentiviral transduction

Lentiviral clones expressing a non-overlapping shRNA against human IL-19 (TRCN58725) and LacZ (TRCN0000231722) were obtained from the National RNAi Core Facility (Academia Sinica, Taiwan). 293 T cells were co-transfected with pLKO.1, pCMV-R8.91, and packaging plasmids (pPAX/pMD.2G) using PolyJet (SignaGen Laboratories, USA). After 72 h, the viruses were concentrated by precipitation with the Speedy Lentivirus Purification Kit (Abcam, USA), following the manufacturer’s instructions. For lentiviral transduction, human GSCs were transduced with lentivirus expressing the shRNA and selected with puromycin for 72 h.

### Bone marrow derived macrophage supernatant effects on CD8^+^ T cell activation

Bone marrow-derived macrophages (BMDMs) were differentiated from WT and *Il-19*^−/−^ bone marrow using M-CSF (100 ng/mL) and then polarized into M2 BMDMs through IL-4 stimulation [38]. CD8^+^ T cells were isolated from the spleens of C57BL/6JNarl mice using a CD8^+^ T cell isolation kit (MACS, USA). M2-enriched BMDM supernatants were collected and used to culture 2 × 10^5^ CD8^+^ T cells in the presence of biotinylated CD3 and CD28 antibodies and anti-biotin MACSiBead particles (MACS, USA) for 3 days. The amount of IFN-γ in the CD8^+^ T cell culture supernatant was measured using an enzyme-linked immunosorbent assay (ELISA) Max Deluxe Mouse IFN-γ Kit (BioLegend).

### Colony formation assay

2000 DBTRG and DBTRG/TMZ-R cells were cultured for 9 days in the presence or absence of 50 μM TMZ. 2000 control (Ctrl) and IL-19KO GL261/TMZ-R cells (KO#1 and KO#2) cells were cultured for 10 days. The cells were then fixed with 4% paraformaldehyde and stained with 0.5% crystal violet. The number of colonies was subsequently counted.

### Cell invasion assay

The invasion abilities of GL261/TMZ-R, U118, and DBTRG/TMZ-R cells were analyzed using transwell chambers (Corning Costar 3422, USA) in accordance with the manufacturer’s protocol. A total of 2000 cells were loaded into Matrigel-coated Transwell upper chambers and incubated with IL-19 (100 ng/mL) in the lower wells, which acted as a chemoattractant. The number of cells per field was counted in five random fields of each membrane under an optical microscope. In some experiments, 1 μg/mL WISP1 antibody (R&D Systems) or isotype control antibody was added to the upper and lower chambers.

### Western blot

The expression levels of pAkt (Ser473), GAPDH, Phospho-β-Catenin Ser552 (Cell Signaling Technology, USA), IL-20RA and IL-20RB (Thermo, USA), and IL-19 and WISP1 (R&D Systems) were determined in cell lysates by western blotting. Cell lysates were prepared using the PRO-PREP protein extraction solution (iNtRON Biotechnology, Korea) with 2 mM Na_3_VO_4_. Horseradish peroxidase-conjugated goat antirabbit, antimouse, or antirat IgG antibody (GoalBio, Taiwan) was used as secondary antibody. All data were acquired using a ChemiDoc Touch Imaging System (Bio-Rad, USA).

### T cell activation assay

T cells were isolated from the spleens of C57BL/6JNarl mice using a Pan T Cell Isolation Kit (MACS, USA). TCs were activated using plate-bound CD3 antibody (2C11, 10 μg/mL) and soluble CD28 antibody (37.51, 2 μg/mL) in the presence or absence of IL-19 (50 ng/mL). After 2 days of incubation, the TCs were stimulated with phorbol myristate acetate (PMA, 100 ng/mL) and an ionophore (A23187, 1 μg/mL) for 3 h. Brefeldin A (5 μg/mL) was added during the last 2 h of culturing. The cells were stained with IFN-γ, CD8, and CD4 antibodies in accordance with standard protocols and then analyzed using flow cytometry.

### Flow cytometry

Cell surface molecules were stained with specific antibodies in accordance with standard protocols and analyzed using a CytoFlex Flow Cytometer (Beckman Coulter, USA). The following fluorochrome-conjugated antibodies were used to detect IL-19 expression in the tumor-infiltrating cells: IL-19-Alexa Fluor 647 (152112), Ly6G-PB (RB6-8C5), CD45-APC-Cy7 (30-F11), CD11b-PE (M1/70), Ly6C-FITC (HK1.4), CD8-PE (53–6.7), and CD4-PE-Cy7 (GK1.5). The antibodies were purchased from BioLegend or R&D Systems.

### Proteomics analysis for GBM cells

First, 1 × 10^7^ GL261 and GL261/TMZ-R cells from each cell line were lysed using 0.5 mL protein extraction buffer (Abcam, USA) with a mass spectrometry-safe protease and phosphatase inhibitor (Sigma, USA). The protein lysates were subsequently reduced and alkylated by adding 2 M urea and 5 mM dithiothreitol (Thermo Scientific, USA) and incubated for 30 min at 37 °C. The mixture was alkylated with 15 mM iodoacetamide (Sigma, USA) for 30 min in the dark at room temperature. The proteins were digested by adding trpsin/LysC (Promega, USA) overnight at room temperature at a 1:50 enzyme-to-protein ratio. The digested samples were acidified with trifluoroacetic acid to achieve a final volumetric concentration of 0.5% and then centrifuged at 15,000 g for 10 min to clear the precipitated urea from the peptide lysates. The samples were dried using a SpeedVac system and desalted using peptide desalting spin columns (Pierce, Thermo Scientific, USA). The peptides were then subjected to reversed-phase fractionation using a high-pH reversed-phase peptide fractionation kit (Pierce, Thermo Scientific, USA) and dried using a SpeedVac system. The dried peptides were desalted using a Ziptip-C18 column (Merck Millipore). Liquid chromatography with tandem mass spectrometry (LC–MS) was performed using a Thermo LTQ Orbitrap Elite mass spectrometer. The peptide mixtures were loaded onto a C18 BEH column 25 cm in length with an inner diameter of 75 μM and packed with 1.7-μM particles with a pore width of 130 Å. The peptides were then separated for 150 min using a segmented gradient from 5 to 35% solvent B (acetonitrile with 0.1% formic acid) at a flow rate of 300 nL/min. The LC–MS raw data were aligned using Progenesis QI software (Waters Corporation), and the proteins were identified using Mascot software.

### Procedures of CHOL-PEG-SPIO-IL19 conjugate synthesis

The CHOL-PEG-SPIO-IL19 nanoparticles were constructed in our previous study [34] with some modifications. Briefly, 0.25 mL of amine-functionalized SPIO nanoparticles (1 mg/mL [Fe] = 0.86 nmol/mL nanoparticles, 10 nm in size; Ocean NanoTech, USA) was reacted with 37.5 μL of sulfo-SMCC (10 mg/mL, 858 nmol) at room temperature for 1 h to obtain maleimide-functionalized SPIO nanoparticles. The maleimide-functionalized SPIO nanoparticles were washed with 10 mL of phosphate-buffered saline (PBS) to remove excess free sulfo-SMCC using an LS column (Miltenyi Biotech, Germany) and then eluted in 800 μL of PBS. Subsequently, human IL-19 antibodies (0.5 mg/mL; R&D Systems, USA) were treated with iminothiolane (1.2 μg, 8.7 nmol; Thermo Fisher Scientific, USA), dissolved in 200 μL of Traut’s reagent (50 mM NaHCO3, 150 mM NaCl, and 10 mM EDTA, pH 8.6), and reacted at room temperature for 60 min. After thiolation, 200 μL of 10 mM tris(2-carboxyethyl)phosphine (TCEP; Sigma-Aldrich, USA) was added at room temperature for 30 min. The solution was replaced with 5 mM EDTA in PBS using Vivaspin (10-kDa MWCO polyethersulfone, Sartorius, USA). Finally, the precursor cholesterol poly(ethylene glycol)-thiol (Cholesterol-PEG-SH, MW 2000, 1 mg/mL; NSP, USA), the thiolated antibodies, and the maleimide-functionalized SPIO nanoparticles were mixed and reacted at 4 °C overnight. The unused maleimide-functionalized groups were blocked with excess cysteine for 15 min at room temperature. The CHOL-PEG-modified IL-19 antibody-conjugated SPIO nanoparticles were separated using an MS column and washed with PBS at a volume 25 times greater than the column bed volume to remove unconjugated antibodies. The nanoparticles were then eluted in 600 μL of PBS. The number of immobilized IL-19 antibodies per SPIO nanoparticle was estimated to be two, based on the molarities of the components in the reaction. The total amount of SPIO in the CHOL-PEG-SPIO-IL19 nanoparticles was determined using a spectrophotometric technique (absorption at 500 nm). The fluorescence and absorption signals were measured using the Thermo Varioskan Flash (Thermo Fisher Scientific, USA) and the Multiskan GO Microplate Spectrophotometer (Thermo Fisher Scientific, USA), respectively. CHOL-PEG-SPIO-isotype control nanoparticles were generated using the same procedures, but the IL-19 antibody was replaced with an isotype control antibody (mouse IgG2B, R&D Systems). Unmodified SPIO nanoparticles were used to generate SPIO-IL-19 nanoparticles and followed the same conjugation procedures.

### Physicochemical characteristics of CHOL-PEG-SPIO-IL19 conjugate

The measurements of hydrodynamic diameter of the SPIO and SPIO conjugate nanoparticles were determined by Zetasizer Nano ZSP (Malvern Instruments, UK), which using a process called Dynamic Light Scattering (DLS) according to manufacturer instructions. The dispersant of SPIO conjugate nanoparticles was 10 mM NaCl solution. The infrared spectrometer of the SPIO, carriers (Cholesterol-PEG and human IL19 antibody), and SPIO conjugate nanoparticles was determined by subjecting the samples to Fourier-Transform Infrared (FT-IR) Spectroscopy using PERKINELMER FRONTIER (Waltham, USA). The measurement of IR spectrum for the disc of each sample over a wavelength scanning range of 4000 cm^−1^ to 400 cm^−1^ to observe the conversion of the functional groups of the copolymer.

To prepare liquid samples containing particles for transmission electron microscopy (TEM), the negative staining technique was employed. Formvar/carbon-coated 200-mesh nickel grids were used as the substrate for sample deposition. A drop of the particle-containing liquid sample was applied onto the grid and allowed to stand for 3 min at room temperature. Excess liquid was carefully removed using filter paper to minimize background interference. Subsequently, the grid was stained with 2% uranyl acetate for 1 min to enhance contrast. After staining, excess uranyl acetate was similarly removed with filter paper. The grid was then air-dried completely before being mounted for TEM observation. TEM images had been acquired by using an HT-7700 scope (Hitachi, Japan), and the analysis was performed according to the manufacturer’s instructions.

To detect the IL-19 expression in human GBM cells, we determined the IL-19 signals through flow cytometry and immunofluorescence assay. The intracellular staining with specific antibodies according to the standard protocols. DBTRG cells were stained with CHOL-PEG-SPIO-IL19 nanoparticles, unconjugated SPIO nanoparticles, IL-19 antibodies (MAB1035, R&D, USA) and isotype control antibodies (402202, BioLegend, USA), followed by FITC-conjugated goat anti-mouse IgG (H + L) antibodies (A-11029, Thermo, USA) by standard protocols and analyzed on a CytoFLEX flow cytometer (Beckman Coulter, USA). Data were analyzed using FlowJo software. The signal of IL-19 expression in DBTRG cells were also determined by immunoflurosence assay with a mounting medium containing 4′6-diamidino-2-dole (DAPI) (Vectashield, USA).

### In vitro CHOL-PEG-SPIO-IL19 phantom analysis

MRI images were obtained using a 7 T Bruker PharmaScan MRI scanner having a volume coil with an inner diameter of 72 mm (Bruker BioSpin, MA, USA). T2-weighted images were acquired using spin-echo sequences with an echo time (TE) of 8 ms, a repetition time (TR) of 3000 ms, 50 echoes, a field of view (FOV) of 50 × 50 mm^2^, a resolution of 256 × 256, and a slice thickness of 1 mm. The MRI samples were CHOL-PEG-SPIO-IL19 nanoparticle phantoms suspended in 1% agarose gel.

### Animal MRI measurements

20,000 human GSC cells were injected into the brain of NBSGW mice. In vivo MRI images of mouse brains were obtained using a 7 T Bruker PharmaScan MRI scanner using a volume coil with an inner diameter of 72 mm (Bruker BioSpin). The MRI scanning protocol was followed our previous study [34]. Briefly, MRI was performed in mice anesthetized using 2% isoflurane in the coronal plane. The MRI protocol included a T1 image (TR, 341.3 ms; TE, 4.5 ms; flip angle, 30°; FOV, 16 × 16 mm^2^; matrix, 256 × 256; 2D; slice thickness, 0.75 mm; number of excitations, 8; resolution, 0.0625 × 0.0625 × 0.75 mm^3^), a T2-weighted image (TR, 2500 ms; TE, 33 ms; flip angle, 45°; FOV, 16 × 16 mm^2^; matrix, 256 × 256; 2D; slice thickness, 0.75 mm; number of excitations, 8; resolution, 0.0625 × 0.0625 × 0.75 mm^3^), a T2*-weighted image (TR, 1000 ms; TE, 12 ms; flip angle; FOV, 16 × 16 mm^2^; matrix, 256 × 256; 2D; slice thickness, 0.75 mm; number of excitations, 2; resolution, 0.0625 × 0.0625 × 0.75 mm^3^), SWI (TR, 39 ms; TE, 51.8 ms; flip angle, 15°; FOV, 50 × 50 mm^2^; matrix, 128 × 128; 3D; slice thickness, 0.5 mm; number of excitations, 3; resolution 0.39 × 0.39 × 0.5 mm^3^), and T2* mapping (16-echo gradient echo sequence; TR,1150 ms; minimum TE, 3.3 ms; ∆TE, 3 ms; flip angle, 80°; FOV, 16 × 16 mm^2^; matrix, 256 × 256; 2D; slice thickness, 0.5 mm; number of excitations, 2; resolution, 0.0625 × 0.0625 × 0.5 mm^3^). The initial T2* mapping scan was performed before injection of CHOL-PEG-SPIO-IL19, SPIO-IL-19, CHOL-PEG-SPIO-isotype ctrl, or SPIO nanoparticles. The second T2* mapping session was started 4 h after injection. The T2* value of each voxel was calculated through exponential fitting performed in-house by using MATLAB (version R2023b, MathWorks, Sherborn, MA, USA). The T2* map was first converted to an R2* relaxivity map by taking its reciprocal. The increase in R2* value (∆R2*) after CHOL-PEG-SPIO-IL19, SPIO-IL-19, CHOL-PEG-SPIO-isotype ctrl, or SPIO injection was calculated using the following formulas: ∆R2* = R2* _4 h_ − R2* _0 h_. R2* values measured in MR images following CHOL-PEG-SPIO-IL19, SPIO-IL-19, CHOL-PEG-SPIO-isotype ctrl, or SPIO administration. The ∆R2* value, which indicates the change in relaxivity due to the local aggregation of CHOL-PEG-SPIO-IL19, SPIO-IL-19, CHOL-PEG-SPIO-isotype ctrl, or SPIO nanoparticles, has a linear relationship with the local CHOL-PEG-SPIO-IL19, SPIO-IL-19, CHOL-PEG-SPIO-isotype ctrl, or SPIO nanoparticle concentration. The coefficient of the linear transformation function between ∆R2* and the local CHOL-PEG-SPIO-IL19, SPIO-IL-19, CHOL-PEG-SPIO-isotype ctrl, or SPIO nanoparticle concentration was estimated through linear regression analysis of agarose phantoms containing various concentrations of CHOL-PEG-SPIO-IL19 nanoparticles. The ∆R2* map was then converted into a local CHOL-PEG-SPIO-IL19, SPIO-IL-19, CHOL-PEG-SPIO-isotype ctrl, or SPIO concentration map by using the estimated linear transformation function. A disk of nickel-coated neodymium iron boron (Nd2Fe14B) with a diameter of 8 mm, a height of 5 mm, and a 0.43-T N42 grade magnet was placed on the tumor site of tumor-bearing mice. After placing the magnet, 50 μg of CHOL-PEG-SPIO-IL19, SPIO-IL-19, CHOL-PEG-SPIO-isotype ctrl, or SPIO nanoparticles was administered via tail vein injection. The magnet was maintained on the tumor site for 1 h and then removed. A radiologist evaluated the MRI images to identify dark signals due to CHOL-PEG-SPIO-IL19, SPIO-IL-19, CHOL-PEG-SPIO-isotype ctrl, or SPIO nanoparticles on T2-weighted images, T2*-weighted images, SWI, and T2* mapping. The tumor volume and hypointense areas in the tumor from T2* and SWI images were calculated using MATLAB. To measure tumor volumes derived from shLacZ and shIL-19 GSCs, T1 MRI images were acquired following administration of the contrast agent Gadovist 1.0 (1 mmol/kg body weight). Post-contrast T1 images were used to delineate tumor boundaries. Tumor margins were manually contoured using the MRIcro software, ensuring precise identification of the enhanced regions corresponding to the tumors. The total tumor volume was calculated by integrating the contoured areas across all relevant image slices using MATLAB, providing a quantitative 3D assessment of tumor size.

### Detection of iron in GBM tissues by prussian blue staining assay

To test the binding of CHOL-PEG-SPIO-IL-19 nanoparticles to tumor tissue, CHOL-PEG-SPIO-IL-19 nanoparticles were detected by Abcam iron stain kit (Abcam, USA). After human GSC tumor-bearing mice were injected with CHOL-PEG-SPIO-IL-19 or CHOL-PEG-SPIO-isotype for 4 h, the brains were fixed and embedded in paraffin and cut into 5-μM-thick sections. Brain sections were then deparaffinized, rehydrated through a graded series of ethanol, then directly subjected to a Prussian blue assay to detect the presence of iron caused by CHOL-PEG-SPIO-IL-19 nanoparticles in the tumor tissue. These sections were observed through microscopy (Olympus/BX43).

### Statistical analysis

This study conducted Unpaired *t*-tests and one-way analyses of variance (ANOVA) were used for all data using GraphPad Prism software. Error bars represent the standard deviation from the means.

## Results

### Clinical characteristics of study cohort

This analysis comprised patients from the TCGA and Taiwan GBM study cohorts. Supplementary Table 2 lists the clinical characteristics of patients with GBM from the TCGA database and the Taiwan GBM study cohort. Most of the patients (86–92%) in the TCGA database had GBM that was associated with poorer overall survival (mean survival days 502–407 days) than *IDH1*-mutant glioma in both the RNA-seq and microarray datasets (mean survival 969–723 days); these results are consistent with those of another study [6]. The mean survival days of patients with IDH1-wt GBM is 571 days in the Taiwan study cohort. Thus, the survival rate of our Taiwan GBM cohort was consistent with that of patients in TCGA GBM dataset.

### High *Il-19* expression was associated with poor survival in patients with GBM

**Fig. 1:**
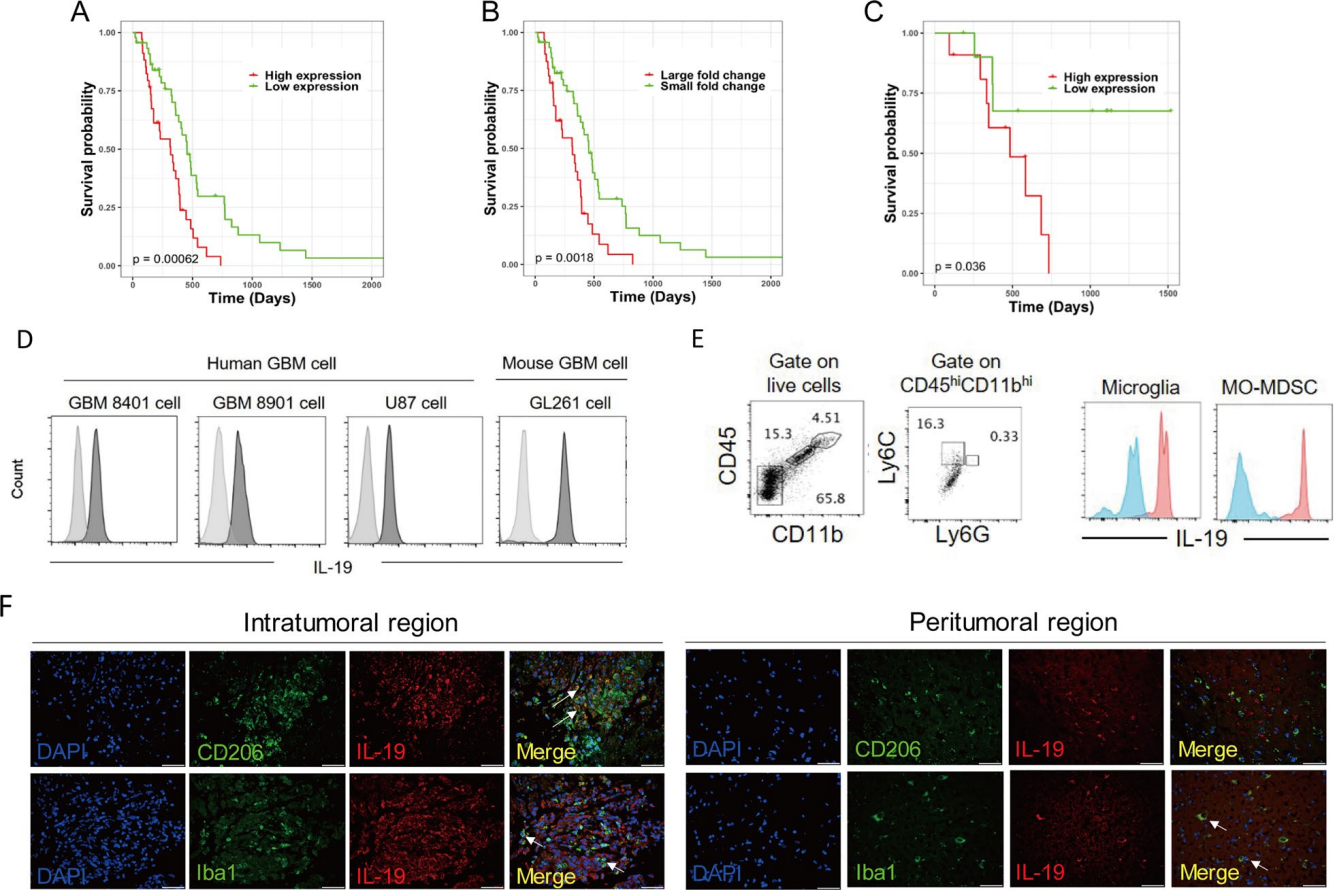
*IL-19* expression in tumor associated with poor prognosis in patients with GBM. **A** Survival probability of patients with GBM in two groups with high (*n* = 34) and low *IL-19* (*n* = 45) expression obtained from RNA-seq data in TCGA database. **B** Survival probability of patients with GBM in two groups with large (*n* = 32) and small (*n* = 47) log_2_ fold change in relative *IL-19* expression to TATA-binding protein expression level observed from RNA microarray data in TCGA database and in **C** large fold change group (*n* = 11) and low fold change group (*n* = 11) in Taiwan GBM study cohort. The *p* values for the log-rank test are indicated in the survival probability graph. **D** IL-19 expression levels in human GBM cell lines (GBM8401, GBM8901, and U87 cells) and murine GBM cell line (GL261) were determined through flow cytometry. Light-gray histogram represents isotype control antibody staining. **E** Flow cytometric gating for tumor-infiltrating microglia (CD45^dim^CD11b^dim^) and MO-MDSCs (CD45^hi^CD11b^hi^Ly6C^+^Ly6G^−^) in GL261 tumor–bearing mice. Blue histogram represents isotype control antibody staining to examine IL-19 expression level in brain-infiltrating lymphocytes. **F** Immunofluorescent staining of the IL-19 (red), M2 TAM Marker CD206 (green) and the pan-macrophage/microglia marker Iba1 (green) in intratumoral and peritumoral region of human GBM tissue. White arrows indicate CD206^+^IL-19^+^ or Iba1^+^IL-19^+^ cells. Scale bar: 50 μM

Given that immune response genes are associated with survival in patients with GBM [62], we analyzed the relationship between the expression of 8,901 immune-related genes and survival in patients with GBM. We excluded patients with *IDH1*-mutant gliomas (Supplementary Table 2) because *IDH1* mutations are associated with favorable survival and are not included in the GBM classification in the 2021 World Health Organization Classification of Tumors of the Central Nervous System [2]. We found that *Il-19* was among the top five immune-related genes strongly associated with poor survival in patients with GBM. Patients with GBM with high *Il-19* expression levels in tumor tissues had lower survival rates than those with low expression levels according to RNA-seq (Fig. 1A) and microarray data (Fig. 1B). Data from the Taiwanese cohort (Fig. 1C) yielded similar results. These findings indicate that *Il-19* is associated with poor prognosis in patients with GBM.

### Expression of IL-19 and its receptors in GBM cells and tumor

To explore the function of IL-19 in GBM, we first examined *Il-19* expression levels in human GBM tissue and its peritumoral region. RNA-seq data indicated that the expression levels of *Il-19*, *Il-20RA*, and *Il-20RB* were higher in GBM tissues than in normal brain tissue (Supplementary Fig. 2A). We obtained peritumoral tissue through MR-guided stereotaxic surgery and performed microarray analysis to investigate the *Il-19* and its receptors expression levels in intratumoral and peritumoral region of human GBM tumor tissue. Microarray analysis revealed that *Il-19* was expressed in both the intratumoral and peritumoral regions and that *Il-20RB* was more highly expressed in the intratumoral region than in the peritumoral region (Supplementary Fig. 2B, 2D), indicating that *Il-19* was expressed in the tumor core and the adjacent peritumoral region of GBM. Next, we identified cell types with high expression of *Il-19* in the GBM microenvironment, which included human and murine GBM cells (GBM8401, GBM8901, U87, and GL261) and two tumor-infiltrating subsets (microglia-like cells and MDSCs) in GL261 tumor-bearing mice (Fig. 1E). To identify the major IL-19-expressing cell types in the intratumoral and peritumoral regions of GBM, we found that most cells in the intratumoral region express IL-19. Additionally, M2-like cells (CD206^+^) and macrophage/microglia (Iba1^+^) also expressed IL-19 in the intratumoral region of tumor tissue from patients with GBM (Fig. 1F). In the peritumoral region, macrophage/microglia cells, but not M2-like cells, expressed IL-19 (Fig. 1F). The expression of IL-19, IL-20RA, and IL-20RB in human GBM cell lines (LN18, DBTRG, and U118) was confirmed by Western blot analysis (Supplementary Fig. 2C). These results indicate that GBM tumor cells, microglia, and MDSCs are sources of IL-19 in GBM tumors.

### High *Il-19* expression is associated with immunosuppressive responses in peritumoral region in patients with GBM

**Fig. 2:**
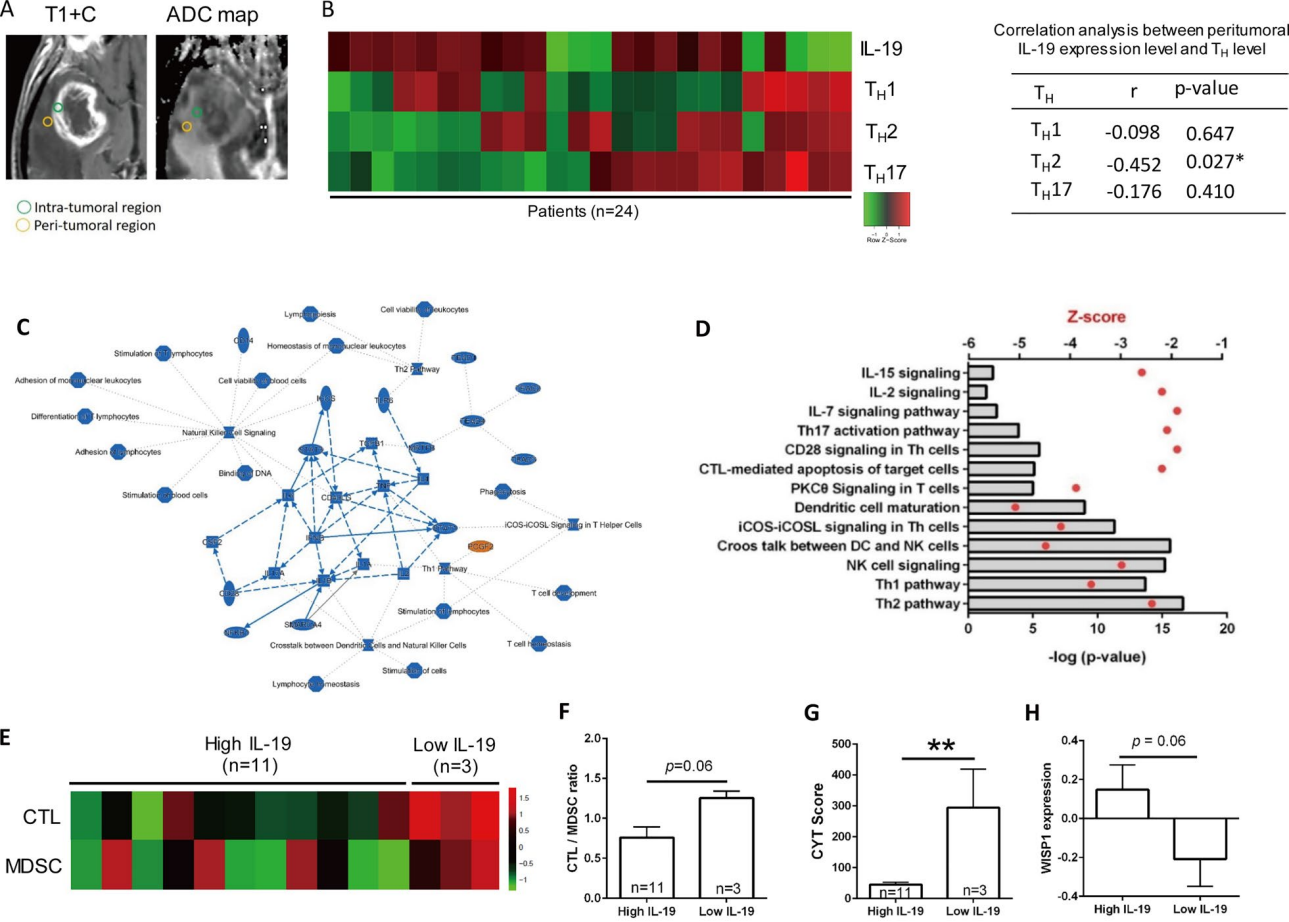
*IL-19* expression is associated with immunosuppressive response in patients with GBM. **A** Site-specific tissue samples, including intratumoral solid T1 contrast-enhanced tissue (T1 + C) and peritumoral region tissue, were collected from patients with GBM in Taiwan through image-guided stereotactic biopsy. Representative T1 + C and apparent diffusion coefficient map (ADC) images of GBM in human brain are presented. **B** Heat map of *IL-19* expression and normalized enrichment level of T_H_ responses in peritumoral region (*n* = 24). Value of correlation coefficient between *IL-19* expression and T_H_ response and *p* values are provided in the table. **C** High *IL-19* expression in peritumoral region was associated with immunosuppressive responses in patients with GBM in Taiwan. Immune-related signaling pathway was predicted using IPA. **D** Bar graph of Z-score and *p* values of predicted immune-related pathways. **E** Heatmap of CTL- and MDSC-normalized enrichment scores in peritumoral region with high and low *IL-19* expression. **F** Ratio of enrichment score of CTL to MDSC and **G** CYT score in peritumoral tissue with high (*n* = 11) and low (*n* = 3) *IL-19* expression. Statistical analysis comprised the Mann–Whitney U test; **p < 0.01. **H** Gene expression value (Z-score transformed) of WISP1 in the tumor tissue with high (*n* = 47) and low IL-19 (*n* = 48) expression from microarray data of TCGA database are shown in the bar graph

T_H_1, T_H_2, and T_H_17 responses are known to be associated with differential prognosis of various cancer types [12]. A T_H_1 response indicates a favorable prognosis in most cancer types, including GBM, where it is considered to have antitumor activity [43]. IL-4, a signature cytokine of T_H_2 cells, can inhibit GBM xenograft growth [14]. T_H_17 exhibit both antitumor [5] and immunosuppressive activity in GBM [52]. Since IL-19 modulates T_H_1, T_H_2, and T_H_17 responses [15, 33], we next investigated whether *IL-19* expression levels can affect these T_H_ responses in the peritumoral and intratumoral regions of patients with GBM (Fig. 2A). RNA microarray analysis revealed that *Il-19* expression levels were negatively correlated with T_H_1, T_H_2, and T_H_17 responses in the peritumoral region (Fig. 2B), but not in the intratumoral region (data not shown), suggesting that IL-19 acts as an immunosuppressive cytokine in the peritumoral region.

To determine whether IL-19 expression levels affect peritumoral immune responses that correlate with patient survival, we examined the peritumoral immune profiles of GBM patients with available survival data by comparing RNA microarray data between peritumoral GBM tissue with high IL-19 expression and low IL-19 expression. IPA analysis demonstrated that high *IL-19* expression in the peritumoral region was associated with the inhibition of immune-related pathways (Fig. 2C; Z-score < 0 and log P < 0), including CTL-mediated apoptosis of target cells, NK cell signaling, and the T_H_1 pathway. These pathways are known to drive antitumor immune responses. Next, we identified the association of immunophenotypes with *Il-19* expression levels in GBM tumors and found that higher *Il-19* expression in the peritumoral region was associated with lower CTL levels (Fig. 2E). Additionally, high *Il-19* expression was associated with a considerably lower ratio of the enrichment score of CTLs to MDSCs (Fig. 2F), as well as a lower cytolytic activity score (CYT) in the peritumoral region (Fig. 2G). These results indicate that high *Il-19* expression is associated with reduced antitumor immunity in the peritumoral region.

### Blocking IL-19 modulates TH cytokines and protumor growth factor expression level and improves survival of GBM-bearing mice

**Fig. 3:**
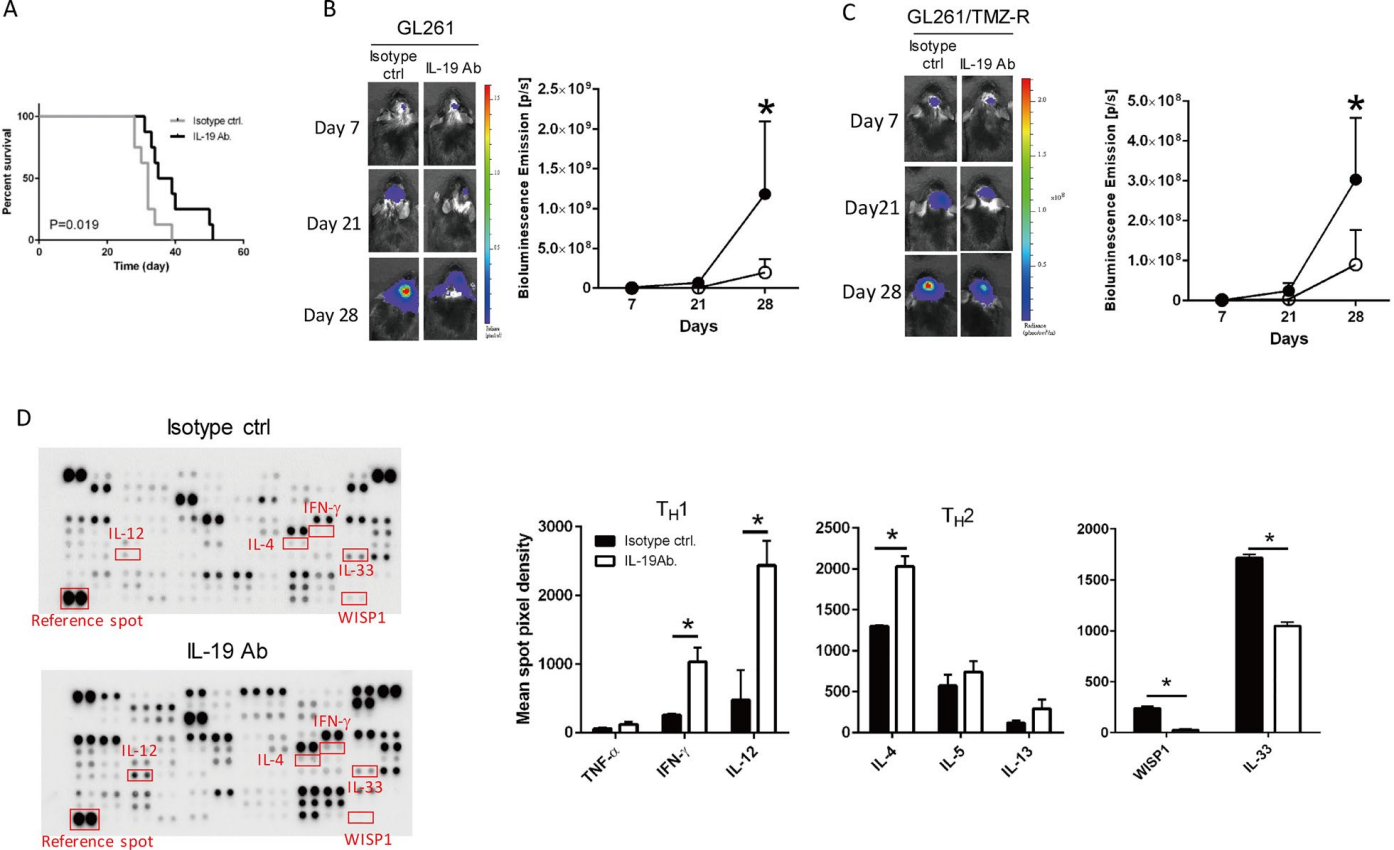
Effects of blocking *IL-19* on TMZ-sensitive and TMZ-resistant GBM progression and cytokine profile in tumor microenvironment. **A** Survival differences in glioma-bearing mice between isotype control groups and IL-19 antibody treatment groups were determined using the Kaplan–Meier method; *n* = 8 in each group. **B** Tumor size in GL261 and **C** GL261/TMZ-R tumor-bearing mice treated with isotype control or IL-19 antibody was determined through IVIS assay; *n* = 8 in each group. **D** GL261 tumor-bearing mice received isotype control antibody or IL-19 antibody treatment. Tumor tissue lysates were harvested on post–tumor inoculation day 28, and cytokine array analysis was performed. Significant differences in T_H_1 and T_H_2 cytokine expression levels between the isotype control antibody and IL-19 antibody treatment groups are indicated by red rectangles. Reference spots indicate that array was incubated with streptavidin–horseradish peroxidase during assay procedure. Quantification of T_H_1-related cytokine (TNF-α, IFN-γ, and IL-12) and T_H_2 cytokine (IL-4, IL-5, and IL-13) expression levels and tumor invasion (WISP1 and IL-33) presented in bar graphs; *p < 0.05

To validate IL-19 as an immunosuppressive cytokine that affects antitumor immunity, as indicated by the results of the human GBM tissue analysis, we applied IL-19 antibody treatment to block IL-19 function in GBM-bearing mice and examined survival outcomes and tumor growth. Mice treated with the IL-19 antibody exhibited improved survival compared with those treated with the isotype control antibody (Fig. 3A). Mice treated with IL-19 antibody inhibited both TMZ-sensitive (GL261) and TMZ-resistant GBM (GL261/TMZ-R) progression (Fig. 3B, C). Cytokine array analysis demonstrated that IL-19 antibody treatment increased the expression of T_H_1-related cytokines (IFN-γ and IL-12) and T_H_2 cytokines (IL-4) and inhibited the expression of tumor invasion-related factors (WISP1 and IL-33) [28, 70] in tumor tissues compared with isotype control antibody treatment (Fig. 3D). RNA microarray analysis also revealed that *WISP1* expression levels were considerably higher in human tumor tissues with high *Il-19* expression than in those with low *Il-19* expression (Fig. 2H). These findings indicate that blocking IL-19 inhibits tumor growth, increases T_H_1 and T_H_2 responses, and reduces tumor invasion–related factors in the tumor microenvironment.

### Functional characterization of the immune clusters in GBM

**Fig. 4:**
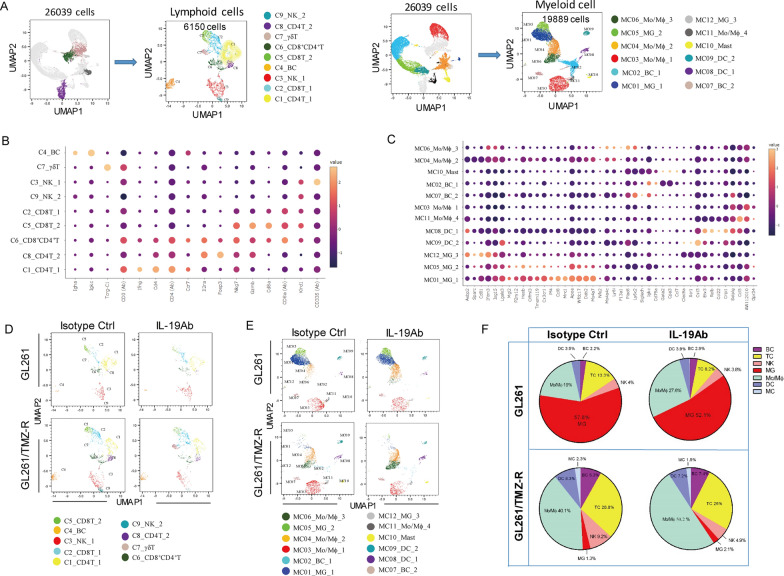
Single-cell transcriptome analysis of tumor-infiltrating immune cells in TMZ-sensitive and TMZ-resistant GBM. **A** UMAP plots of lymphoid and myeloid cells from tumor-infiltrating leukocytes and microglia. A total of 26,039 tumor-infiltrating CD45^+^ cells were harvested on post–tumor inoculation day 28, and scRNA-seq analysis was performed. A total of 6,150 cells from four lymphoid clusters were divided into nine lymphoid clusters (C1–C9). A total of 19,889 tumor-infiltrating CD45^+^ cells from 10 myeloid clusters were divided into 12 clusters (10 myeloid clusters and two B cell clusters). **B** Bubble plot map of expression patterns of selected marker genes across indicated lymphoid clusters and **C** myeloid clusters. Color scale depicts expression level. **D** UMAP plots of lymphoid and **E** myeloid cells from GL261 and GL261/TMZ-R GBM-bearing mice treated with isotype control antibody or IL-19 antibody. **F** Proportions of B cells (BCs), TCs, NK cells, microglia (MG), monocytes/macrophages (Mo/Mϕ), DCs, MCs in GL261 and GL261/TMZ-R treated with isotype control or IL-19 antibody

Our human GBM tumor tissue and cytokine array analysis indicate that IL-19 act as an immunosuppressive cytokine in tumor, however, the mechanism of IL-19 in modulation of immune response in GBM remains unclear. Thus, we performed scRNA-seq analysis to examine whether blocking IL-19 can affect immune profile in GBM. A total of 26,039 CD45^+^ tumor-infiltrating leukocytes were analyzed and 14 cell clusters were identified (4 lymphoid clusters and 10 myeloid clusters; Fig. 4A). To precisely define lymphoid and myeloid clusters, we computationally separated lymphoid cells (6,150 cells) and myeloid cells (19,889 cells) for further analysis. We then identified nine lymphoid clusters (C1–C9) and 12 immune clusters (MC01–MC12), which included two B cell clusters and 10 myeloid clusters (Fig. 4A). Manual annotation based on previously identified marker genes [50] revealed two CD4^+^ TC clusters (C1, C8), two CD8^+^ TC clusters (C2, C5), two NK cell clusters (C3, C9), one γδ TC, one CD8^+^CD4^+^ TC, and one B-cell cluster. (Fig. 4B). Myeloid cluster annotation revealed three microglial clusters (MC01, MC05, and MC12), four monocyte/macrophage clusters (MC03, MC04, MC06, and MC11), two dendritic cell (DC) clusters (MC08 and MC09), and one mast cell (MC) cluster (MC10; Fig. 4C). The top differentially expressed gene profiles of lymphoid and myeloid cell clusters are presented in Supplementary Fig. 3A and B, respectively. Since MC02, MC07, MC11, MC12, C4, and C9 clusters comprised less than 1% of the total immune cells in either GL261 or GL261/TMZ-R tumor, we excluded these clusters from further analysis. To further understand the functional features of these clusters, we utilized the up-regulated DEG profiles to explore the biological pathways enriched in the immune clusters by Metascape analysis, revealing distinct functional associations for each cluster (Table 1 and 2). Metascape analysis demonstrated that the microglia clusters (MC01_MG_1, MC05_MG_2) and monocyte/macrophage clusters (MC03, MC04, MC06) exhibited functional terms generally associated with macrophages, including inflammatory response, positive regulation of response to external stimuli, and type II interferon-γ signaling (Table 1, supplementary Fig. 4). Regarding DC characterization, DC clusters (MC08_DC_1 and MC09_DC_2) were associated with leukocyte activation, cytokine signaling, and regulation of cytokine production (Table 1). For T cell characterization, the results showed that CD4 T cell clusters (C1_CD4T_1 and C8_CD4T_2) are associated with cytokine receptor cytokine-cytokine receptor interaction, and regulation of leukocyte activation. CD8 T cell clusters (C2_CD8T_1, C5_CD8T_2) were associated with adaptive immune response, granzyme-mediated programmed cell death signaling. A CD8^+^CD4^+^ T cell cluster (C6_ CD8^+^CD4^+^T) was associated with mitotic cell cycle, and granzyme-mediated programmed cell death. A γδT cell cluster (C7_γδT) was associated with cellular response to interferon-beta and Th cell differentiation (Table 2, supplementary Fig. 5).

**Table 1:**
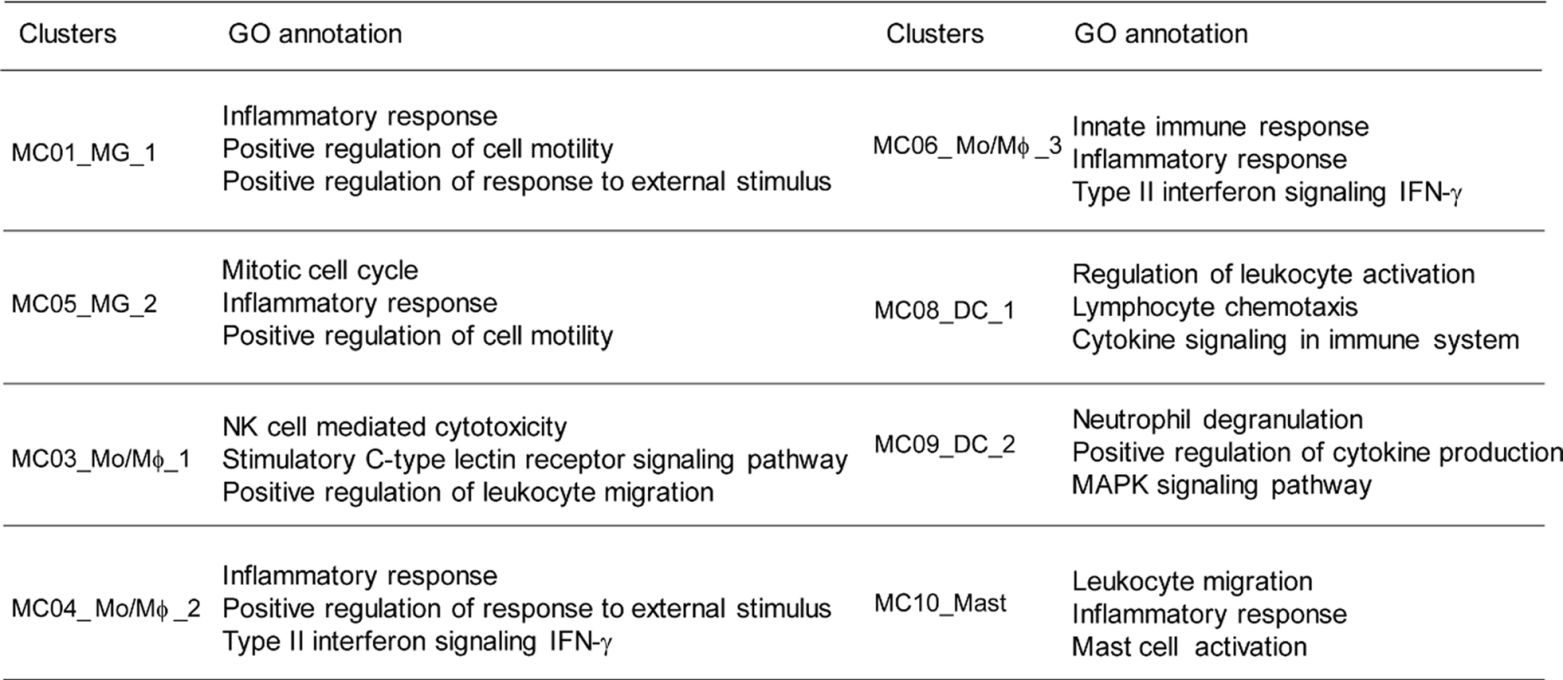
GO annotation of unsupervised clusters in myeloid subsets

**Table 2:**
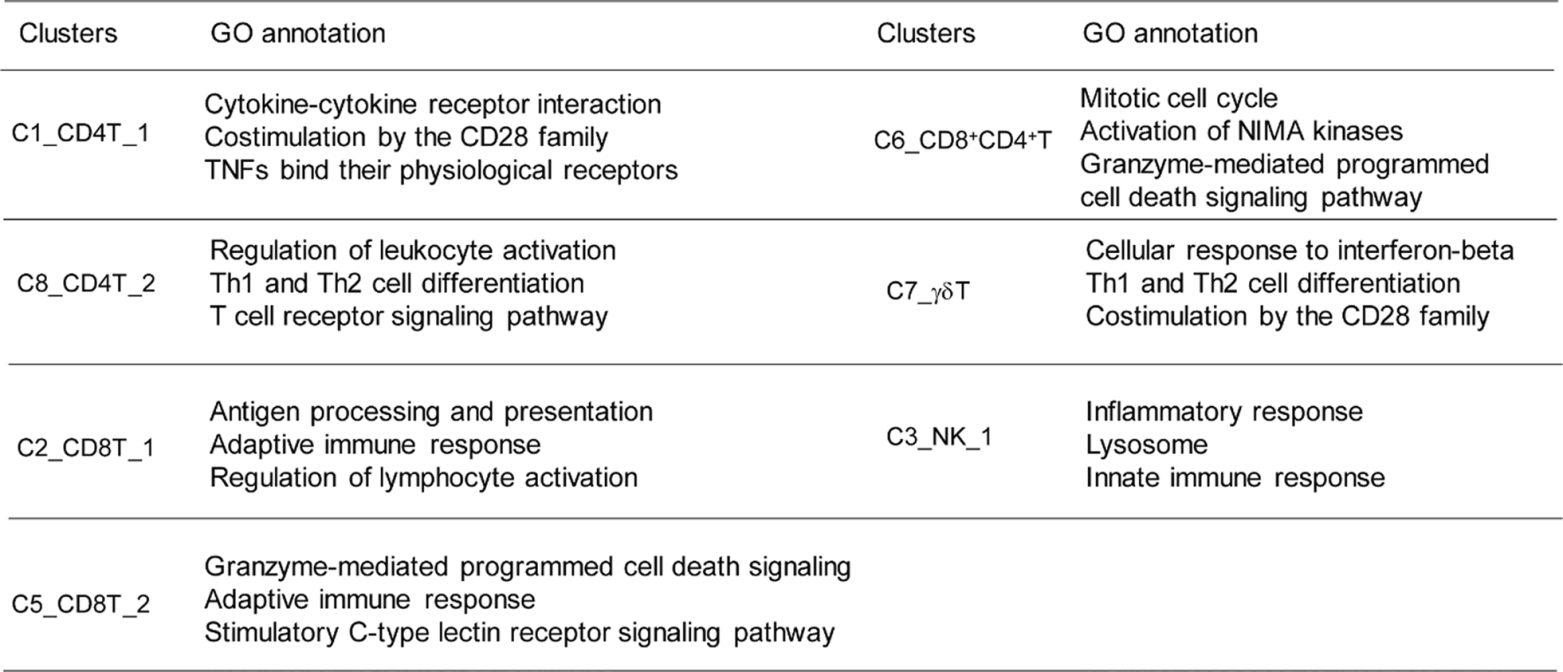
GO annotation of unsupervised clusters in lymphoid subsets

### Blocking IL-19 reprograms the immune cluster composition in TMZ-sensitive and TMZ-resistant GBM

**Fig. 5:**
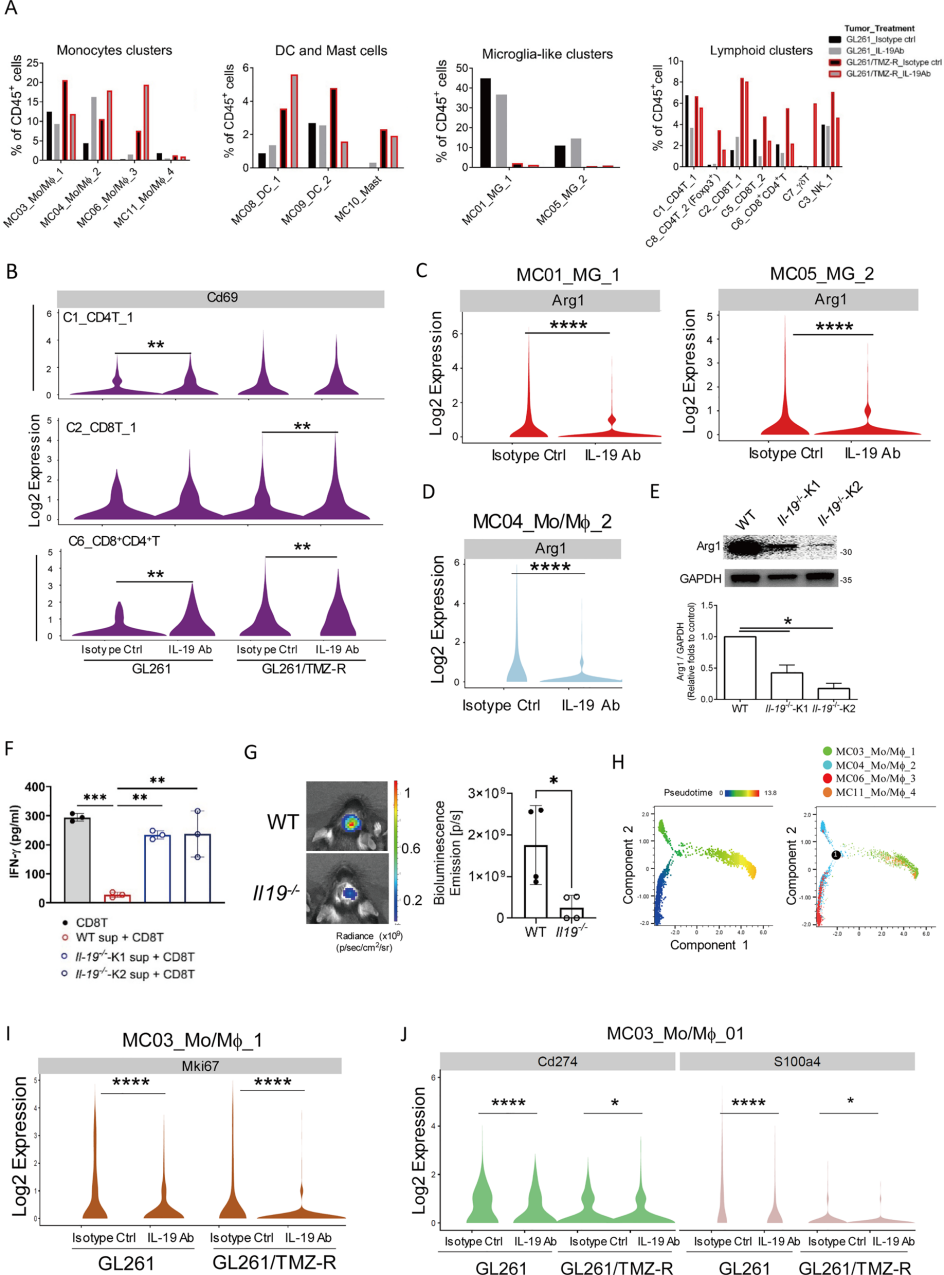
Blocking IL-19 reprogrammed GBM tumor-infiltrating monocyte/macrophage differentiation and disrupted Arg1-mediated CD8 T cell suppression. **A** Percentage of each cluster in tumor-infiltrating CD45^+^ cells from GL261 and GL261/TMZ-R GBM-bearing mice treated with isotype control antibody or IL-19 antibody. **B** Violin plot analysis of *Cd69* expression levels in T cell clusters (C1, C2, and C6) in GL261 and GL261/TMZ-R tumor-bearing mice treated with isotype control or IL-19 antibody; Wilcoxon test; **p < 0.01. **C** Violin plot analysis of *Arg1* expression in microglia (MC01_MG_1, MC05_MG_2). **D** Violin plot analysis of *Arg1* expression in the TAM-like subset. Wilcoxon test; ****p < 0.0001. **E** Expression of Arg1 in BMDMs from WT and IL-19KO mice (*Il-19*^−/−^ K1 and *Il-19*^−/−^ K2) analyzed by Western blot. **F** Effect of *Il-19*^−/−^ M2-like BMDM supernatant on CD8^+^ T cell activation. WT and *Il-19*^−/−^ M2-like BMDM supernatants were used to culture CD8^+^ T cells for 72 h in the presence of antibiotin microbeads and biotinylated CD3 and CD28 antibodies. IFN-γ levels produced by CD8^+^ T cells were determined by ELISA. **G** GL261 tumor progression in WT and *Il-19*^−/−^ mice, determined by IVIS imaging on post-tumor inoculation day 28; n = 4 in each group. *p < 0.05. **H** Differentiation trajectories of tumor-infiltrating macrophage/monocyte subpopulations. **I** Expression of the proliferation marker Ki67 and **J** Immunosuppressive genes (*Cd274* and *S100a4*) in tumor-infiltrating macrophages/monocytes (MC03_Mo/Mϕ_1); Wilcoxon test; *p < 0.05, ****p < 0.0001

To investigate the effect of IL-19 blockade on modulating immune responses in TMZ-sensitive and TMZ-resistant GBM, we first compared the immune profiles between these tumor types. We found that TMZ-resistant tumors contained more macrophages, T cell subsets (C2_CD8T_1 and Foxp3^+^C8_CD4T_2), and mast cells, but fewer microglia-like cells than TMZ-sensitive tumors (Figs. 4D–F, 5A). This suggests that TMZ-resistant GBM is enriched with more TAMs and regulatory T cell (Treg)-like cell subsets. To further elucidate the effects of IL-19 blockade in the tumor microenvironment, we examined the percentages of immune cell subpopulations in GBM-bearing mice after IL-19 antibody treatment. The treatment increased the percentages of a DC subset (MC08_DC_1) and macrophage/monocyte clusters (MC04 and MC06) (Fig. 5A), indicating that blocking IL-19 enhances leukocyte activation and is associated with IFN-γ signaling (Table 1). A Treg-like subset (C8_CD4T_2), which is enriched in TMZ-resistant tumors, was decreased, while a γδ T cell cluster with antitumor activity [29] was significantly increased in IL-19 antibody-treated GL261/TMZ-resistant tumor-bearing mice. This suggests that blocking IL-19 can modulate the proportions of Tregs and γδ T cells in TMZ-resistant GBM. In conclusion, blocking IL-19 alters the immune cluster composition in both TMZ-sensitive and TMZ-resistant GBM, and is associated with IFN-γ-related immune responses.

### Blocking IL-19 upregulates T cell activation in tumors

Since our scRNA data reveal that IL-19 blockade increased the MC04 macrophage and MC08_DC_1 clusters, both of which are associated with IFN-γ signaling and T cell activation, the activation and effector function of T cell subsets could be modulated in the tumors of IL-19 antibody-treated mice. Our results demonstrated that blocking IL-19 indeed promoted T cell activation, as evidenced by violin plots showing higher *Cd69* expression in the CD4^+^ T cell cluster (C1_CD4T_1) and the CD8^+^CD4^+^ T cell cluster (C6_CD8^+^CD4^+^ T) in TMZ-sensitive GBM-bearing mice treated with the IL-19 antibody compared to those treated with the isotype control antibody (Fig. 5B). *Cd69* expression levels were also elevated in the CD8^+^ T cells (C2_CD8T_1) and CD8^+^CD4^+^ T cell clusters in TMZ-resistant GBM-bearing mice treated with IL-19 antibody (Fig. 5B). To further investigate whether IL-19 acts as an immune suppressive gene for CD8^+^ T cell, we used CRISPR/Cas9-mediated genome editing to silence *Il-19* in GL261/TMZ-R cells (IL-19KO GL261/TMZ-R cells), and co-cultured Ctrl or IL-19KO GL261/TMZ-R cells with CD8^+^ T cells undergoing T cell receptor activation. IFN-γ levels were significantly higher in the IL-19KO GL261/TMZ-R cells co-cultured with CD8^+^ T cells (KO + CD8T) compared to the control GL261/TMZ-R group (Ctrl + CD8T; Supplementary Fig. 6A). Additionally, IL-19 treatment inhibited IFN-γ production in both CD8^+^ and CD4^+^ T cells under T cell activation conditions (Supplementary Fig. 6B). Taken together, these results indicate that blocking IL-19 promotes T cell activation in tumors.

### Silencing *Il-19* weakens macrophage suppressive activity on T cell activation and slows tumor progression

Metascape analysis revealed that microglia-like subsets (MC01_MG_1 and MC05_MG_2) are associated with the regulation of immune effector processes and leukocyte-mediated immunity, while TAM-like subsets (MC04_Mo/MΦ_2) are linked to the negative regulation of the immune system (Supplementary Fig. 4). This suggests that these subsets play a regulatory role in antitumor responses. Given that IL-19 stimulation upregulates Arg1 expression in BMDMs [72], we hypothesized that IL-19 might promote Arg1 expression in TAMs. Violin plot analysis demonstrated that *Arg1* expression in both microglia-like subsets (MC01_MG_1 and MC05_MG_2) and the TAM-like subset (MC04_Mo/MΦ_2) was significantly downregulated in IL-19 antibody-treated mice (Fig. 5C, D), suggesting that IL-19 blockade reduces TAM-mediated Arg1-driven immune suppression.

To further explore IL-19’s effect on TAM function, we generated *Il-19*^−/−^ mice (Supplementary Fig. 7) and used their bone marrow to differentiate into Arg1-expressing M2-like BMDMs, which mimic the Arg1-expressing TAMs. The results showed that *Il-19*^−/−^ M2-like BMDMs had lower Arg1 expression compared to WT BMDMs (Fig. 5E), suggest that *Il-19*^−/−^ BMDMs have reduced Arg1-mediated suppression activity. To validate this hypothesis, we collected the culture supernatants of WT and *Il-19*^−/−^ M2 BMDMs and used them to culture CD8^+^ T cells under T cell activation conditions. The results demonstrated that the supernatant from WT M2 BMDMs significantly inhibited IFN-γ production by CD8^+^ T cells, whereas the supernatant from *Il-19*^−/−^ M2 BMDMs lost its inhibitory effects on IFN-γ production from activated CD8^+^ T cells (Fig. 5F). Finally, to assess whether host’s *Il-19* affects GBM progression, we observed that the GL261 tumor burden was significantly lower in *Il-19*^−/−^ mice compared to WT mice 21 days after tumor inoculation (Fig. 5G), indicating that host IL-19 promotes GBM progression. In conclusion, silencing IL-19 weakens the immunosuppressive activity of TAMs and slows tumor progression in GBM.

### Blocking IL-19 modulates immunosuppressive monocytes/macrophages differentiation in tumor microenvironment

To further analyze the temporal dynamics of macrophage subset remodeling, Seurat-defined clusters were superimposed on a pseudo-time trajectory constructed using the Monocle algorithm, and the results revealed that the root of the trajectory was primarily populated by MC06_Mo/Mϕ_3 and differentiated into MC04_Mo/Mϕ_2 or MC03_ Mo/Mϕ_1 (Fig. 5H). IL-19 antibody treatment increased the percentages of MC04_Mo/Mϕ_2 and MC06_Mo/Mϕ_3 in both TMZ-sensitive and TMZ-resistant GBM cells (Fig. 5A), suggest that blocking IL-19 promoted the Mo/Mϕ subsets differentiation into MC04_Mo/Mϕ_2 and MC06_ Mo/Mϕ_3 cells, which are associated with anti-tumor IFN-γ signaling (Table 1). Lastly, IL-19 antibody treatment reduced the percentage of the dominant MC03_Mo/Mϕ_1 subset (Fig. 5A), proliferative gene *ki67* (Fig. 5I), and STAT3-regulated immunosuppressive genes (*Cd274* and *S100a4* [1, 41, 44]) expression level in dominant MC03_ Mo/Mϕ_1 (Fig. 5J), suggesting that blocking IL-19 inhibited immunosuppressive macrophage proliferation and its effector function.

### IL-19 promotes GBM migration and invasion via AKT/β-catenin/WISP1 signaling.

**Fig. 6:**
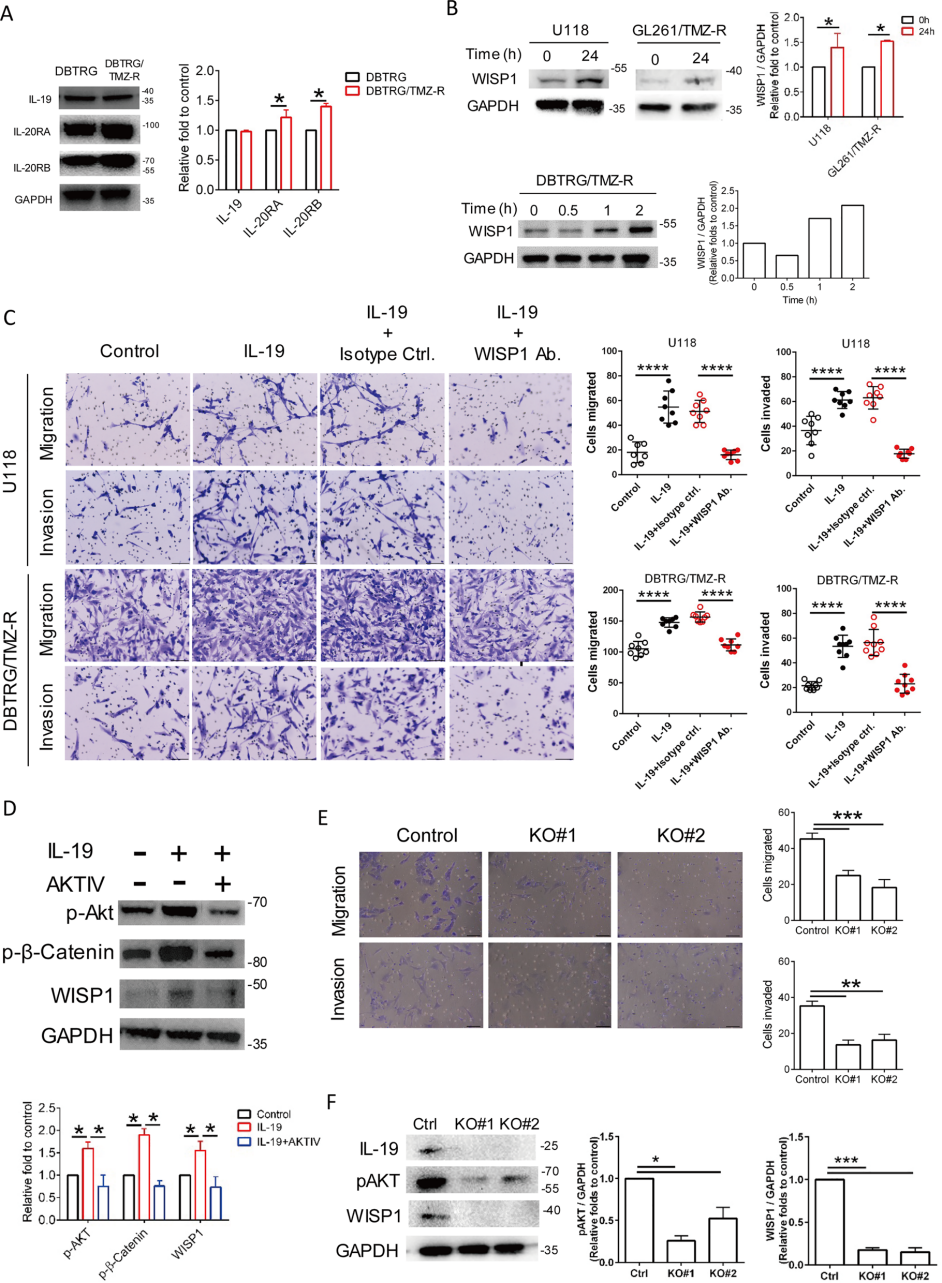
Effects of IL-19 on TMZ-resistant GBM migration and invasion. **A** Expression levels of IL-19, IL-20RA, and IL-20RB in DBTRG and DBTRG/TMZ-R cells were analyzed by Western blot. **B** Human GBM cell (U118) and TMZ-resistant cells (GL261/TMZ-R, DBTRG/TMZ-R) were treated with IL-19 (100 ng/mL) for various duration and WISP1 expression was analyzed through Western blotting. **C** Effect of blocking WISP1 on IL-19-induced GBM cell migration and invasion. U118 and DBTRG/TMZ-R cells were treated with IL-19 (100 ng/mL) in the presence of isotype ctrl. or WISP1 antibodies (1 μg/mL) for 24 h, and invasion was analyzed through a Matrigel-coated transwell migration assay;* n* = 8, one-way ANOVA; ****p < 0.0001. Scale bar: 50 μM. **D** The effect of AKT inhibitor IV (1 μM) on IL-19/WISP1 signaling pathway of U118 cells in the presence of absence of IL-19 (100 ng/mL) for 2 h. Expression level of pAKT, p-β-catenin, WISP1, and GAPDH were determined by Western blot. **E** Effects of *Il-19* KO on migration and invasion of GL261/TMZ-R cells. Migration and invasion ability of Ctrl and two *Il-19* KO clones (KO#1 and KO#2) were determined through Matrigel-coated transwell migration assay; *n* = 3, one-way ANOVA; **p < 0.01, ***p < 0.001. **F** IL-19, pAKT, WISP1, and GAPDH expression levels in Ctrl and *IL-19* KO GL261/TMZ-R cells were determined by Western blot

To determine whether IL-19 and its receptor expression are specifically associated with the acquisition of TMZ resistance in GBM, we generated a TMZ-resistant DBTRG cell line (DBTRG/TMZ-R) through a long-term incubation with TMZ. We found that TMZ-resistant DBTRG cells expressed higher levels of IL-20RA and IL-20RB, but not IL-19, compared to parental DBTRG cells (Fig. 6A), suggesting that GBM cells may respond more to IL-19 stimulation after acquiring TMZ resistance.

Since our cytokine array data showing IL-19 blockade downregulated WISP1 expression (Fig. 3D), and this result is consistent with the observation that WISP1 expression is higher in human GBM tissues with high *Il-19* expression compared to those with low *Il-19* expression (Fig. 2H), suggesting a novel regulatory role of IL-19 in WISP1 expression in GBM. WISP1 is a secreted cysteine-rich protein belonging to the CCN (CYR61, CTGF, and NOV) family and is a target gene of the Wnt/β-catenin pathway [39]. To validate the regulatory link between IL-19 and WISP1 expression, we found that IL-19 treatment upregulated WISP1 expression in the human GBM cell line U118 and in TMZ-resistant GBM cells (GL261/TMZ-R, DBTRG/TMZ-R) (Fig. 6B). Since WISP1 is an oncogene that promotes GBM proliferation and invasion [28], we next validated whether IL-19 could positively regulate GBM invasion via WISP1 signaling. Our data demonstrated that IL-19 promotes cell migration and invasion through WISP1 signaling, as blocking WISP1 with antibodies significantly inhibited IL-19-mediated invasion in GBM cells (U118 and DBTRG/TMZ-R) (Fig. 6C). Since AKT drives phosphorylation of β-catenin [11] which can activate the WISP1 oncogenic pathway, we hypothesized that IL-19 promotes WISP1 expression via AKT phosphorylation. Western blot data confirmed that IL-19 promoted the phosphorylation of AKT and β-catenin, while this phosphorylation was inhibited by an AKT VI inhibitor in U118 cells (Fig. 6D). These findings indicate that IL-19 triggers the AKT/β-catenin/WISP1 axis in GBM cells. To further validate the intrinsic role of the IL-19/WISP1 axis in GBM invasion, we used CRISPR/Cas9-mediated genome editing to silence *Il-19* in GL261/TMZ-R cells (two *Il-19* KO clones: KO#1 and KO#2) since GL261/TMZ-R cells exhibited higher migration and invasion activity than parental GL261 cells by proteomic analysis (Supplementary Fig. 8). Our data revealed that *Il-19* KO cells displayed significantly reduced migration and invasion capabilities (Fig. 6E) as well as markedly lower expression of IL-19, pAKT, and WISP1 (Fig. 6F). In conclusion, these findings indicate that IL-19 promotes GBM migration and invasion via the IL-19/AKT/WISP1 axis.

### Silencing tumor-derived IL-19 inhibits clonogenic growth and tumor progression

IL-19 has been implicated in promoting breast cancer cell proliferation; however, its influence on glioblastoma cell proliferation remains ambiguous. We conduct colony formation assay and found that silencing *IL-19* in GL261/TMZ-R cells (two *IL-19*KO clones, KO#1 and KO#2) substantially reduced the number of colonies compared with the control GL261/TMZ-R cell line (Ctrl) (Supplementary Fig. 9A). This result indicates that silencing IL-19 inhibits clonogenic survival in TMZ- resistant GBM cells. Whether tumor cell-derived IL-19 in driving in vivo tumor progression remains unaddressed. Thus, we established a human GBM tumor-bearing mouse model using immunocompromised NBSGW mice. Initially, we evaluated various human GBM cell lines, including U118 and DBTRG; however, these lines failed to establish tumors in the brains of NBSGW mice (data not shown). In contrast, human GSCs successfully formed tumors in these mice. Thus, we knocked down IL-19 in GSCs and found that the tumor volume of shIL-19 GSC-derived tumors was smaller than that of shLacZ tumors 13 days post-GSC inoculation (Supplementary Fig. 9B). The number of Ki67^+^ cells in shIL-19 GSC-derived tumors was less than that in shLacZ tumors (Supplementary Fig. 9C), suggesting that tumor-derived IL-19 positively regulates tumor cell proliferation.

### Synthesis and characterization of CHOL-PEG-SPIO-IL19

**Fig. 7:**
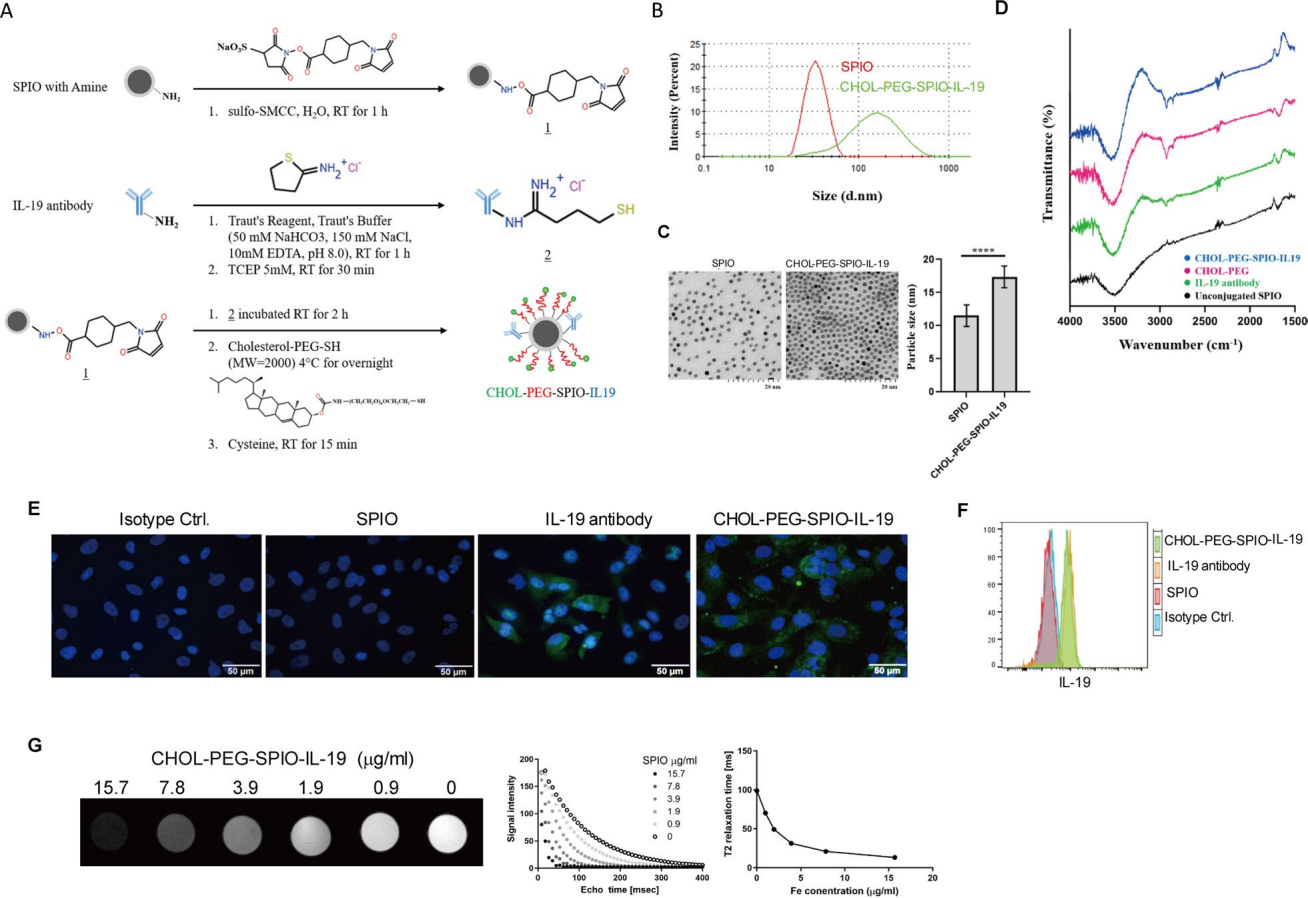
CHOL-PEG-SPIO-IL19 Synthesis process and characterization in vitro. **A** Synthetic scheme of IL-19 antibodies and CHOL-PEG polymer conjugated with amino-coated SPIO nanoparticles. 1. Amino-coated SPIO nanoparticles were modified with maleimide through reaction with sulfo-SMCC. 2. IL-19 antibodies were thiolated with iminothiolane, and TCEP was added to reduce disulfide bonds. 3. The maleimide-functionalized SPIO nanoparticles were mixed with the thiolated antibody solution and CHOL-PEG polymer to form CHOL-PEG and antibody-conjugated SPIO nanoparticles. **B** The size distributions of CHOL-PEG-SPIO-IL19 and unconjugated SPIO nanoparticles were determined through DLS measurement, as shown on the volume distribution graph. **C** TEM images of unconjugated SPIO and CHOL-PEG-SPIO-IL19 (scale bar = 20 nm). Statistical analysis indicated a significant difference among the particle size of CHOL-PEG-SPIO-IL19 and SPIO nanoparticles (n = 10 per group). **D** FT-IR spectrum of CHOL-PEG, IL-19 antibody, CHOL-PEG-SPIO-IL19 and unconjugated SPIO nanoparticles. The IR spectrum of Cholesterol-PEG polymer is characterized by peak at approximately 2854 cm^−1^ due to C-H stretching. **E** Evaluation of CHOL-PEG-SPIO-IL19 nanoparticles targeting specificity through IF staining. DBTRG cells were incubated with IL-19 antibodies, isotype control antibodies (mouse IgG2b antibody), SPIO nanoparticles, and CHOL-PEG-SPIO-IL19 nanoparticles, and then stained with FITC goat anti-mouse IgG2b antibodies. Green, IL-19; blue, DAPI. **F** Flow cytometry analysis for intracellular IL-19 expression in human GBM cells by using CHOL-PEG-SPIO-IL19 nanoparticles. DBTRG cells were stained with SPIO nanoparticles, CHOL-PEG-SPIO-IL19 nanoparticles, IL-19 antibodies, or isotype control antibodies (mouse IgG2b antibody) and then stained with FITC goat anti-mouse IgG2b antibodies. **G** Detection of CHOL-PEG-SPIO-IL19 in T2-weighted MRI images. Echo time curve fitting of CHOL-PEG-SPIO-IL19 phantoms in various known concentrations was measured using 7 T MRI. T2 relaxation time of CHOL-PEG-SPIO-IL19 in known Fe concentrations (n = 3 for each concentration). Corresponding signal intensity images of CHOL-PEG-SPIO-IL19 standards (0–15.7 μg/mL) measured using T2-weighted images

To generate an MRI contrast agent with targeting specificity for human IL-19 in GBM, we developed novel CHOL-PEG-modified SPIO nanoparticles conjugated with IL-19 antibodies as a potential biodegradable brain drug delivery system. The synthesis procedure of CHOL-PEG-SPIO-IL19 nanoparticles is illustrated in Fig. 7A. The particle size distribution of CHOL-PEG-SPIO-IL19 and unconjugated SPIO samples was measured by DLS. The hydrodynamic diameter of the unconjugated SPIO nanoparticles was 32.1 ± 0.2 nm. When SPIO was conjugated with CHOL-PEG and IL-19 antibodies, the hydrodynamic diameter of the CHOL-PEG-SPIO-IL19 nanoparticles increased to 121.4 ± 0.8 nm (Fig. 7B). The morphology of both SPIO and CHOL-PEG-SPIO-IL19 nanoparticles was determined by TEM, showing that both types of nanoparticles were spherical in shape and evenly dispersed. The diameters of CHOL-PEG-SPIO-IL19 nanoparticles were larger than those of unconjugated SPIO (Fig. 7C). Additionally, FT-IR results demonstrated a PEGylated surface on the CHOL-PEG-SPIO-IL19 nanoparticles, with absorption bonds of CHOL-PEG emerging at wavenumber 2854 cm^−1^ due to the C-H stretching vibration peak (Fig. 7D). Taken together, these data confirm that CHOL-PEG polymers were successfully modified onto the surface of SPIO nanoparticles.

### Evaluation of the targeting specificity of CHOL-PEG-SPIO-IL19 to IL-19-expressing GBM cells

To evaluate the IL-19 targeting specificity of CHOL-PEG-SPIO-IL19, we determined that the amount of SPIO in the conjugate nanoparticles was 0.03 μg/μl, as measured by UV–VIS. Next, we used CHOL-PEG-SPIO-IL19 to detect IL-19 expression in the human GBM cell line DBTRG. The immunofluorescence assay demonstrated that positive signals in DBTRG cells could be detected using both CHOL-PEG-SPIO-IL19 nanoparticles and IL-19 antibodies (Fig. 7E). Flow cytometry analysis produced consistent results, showing positive fluorescence signals in DBTRG cells stained with CHOL-PEG-SPIO-IL19 nanoparticles, with fluorescence levels similar to those in the IL-19 antibody-stained group (Fig. 7F). In contrast, no significant signal was detected for the unconjugated SPIO nanoparticle control or the isotype control antibody. These data indicate that IL-19 antibodies retained their affinity for IL-19 after conjugation with CHOL-PEG-SPIO nanoparticles and can detect IL-19 in GBM cells in vitro.

### MR fitting curve and T2 relaxation time of CHOL-PEG-SPIO-IL19 phantoms

To evaluate the feasibility of using CHOL-PEG-SPIO-IL19 for detecting IL-19-positive cells in MRI, phantom scans of CHOL-PEG-SPIO-IL19 were performed to assess the MRI signal intensity and its response to T2 relaxation time at various concentrations. The signal intensity and T2 relaxation time of CHOL-PEG-SPIO-IL19 suspended in agarose gel with known Fe concentrations (0–15.7 μg/mL) were measured using a 7 T MRI system. The T2-fitting curve of CHOL-PEG-SPIO-IL19 demonstrated a correlation between signal intensity and nanoparticle concentration at TE values ranging from 16 to 376 ms (Fig. 7G). Additionally, CHOL-PEG-SPIO-IL19 showed a decrease in MR T2 relaxation time as the Fe concentration increased (Fig. 7G). Specifically, the T2 relaxation time was 71.1 ms at 0.98 μg/mL of Fe, decreasing to 13.1 ms at 15.7 μg/mL. In summary, the MRI signal intensities of various concentrations of CHOL-PEG-SPIO-IL19 phantoms were positively correlated with dark signals in T2-weighted in vitro MR images, suggesting that CHOL-PEG-SPIO-IL19 can be quantitatively detected using in vitro MRI.

### Detection of IL-19 in human GBM through in vivo MRI

**Fig. 8:**
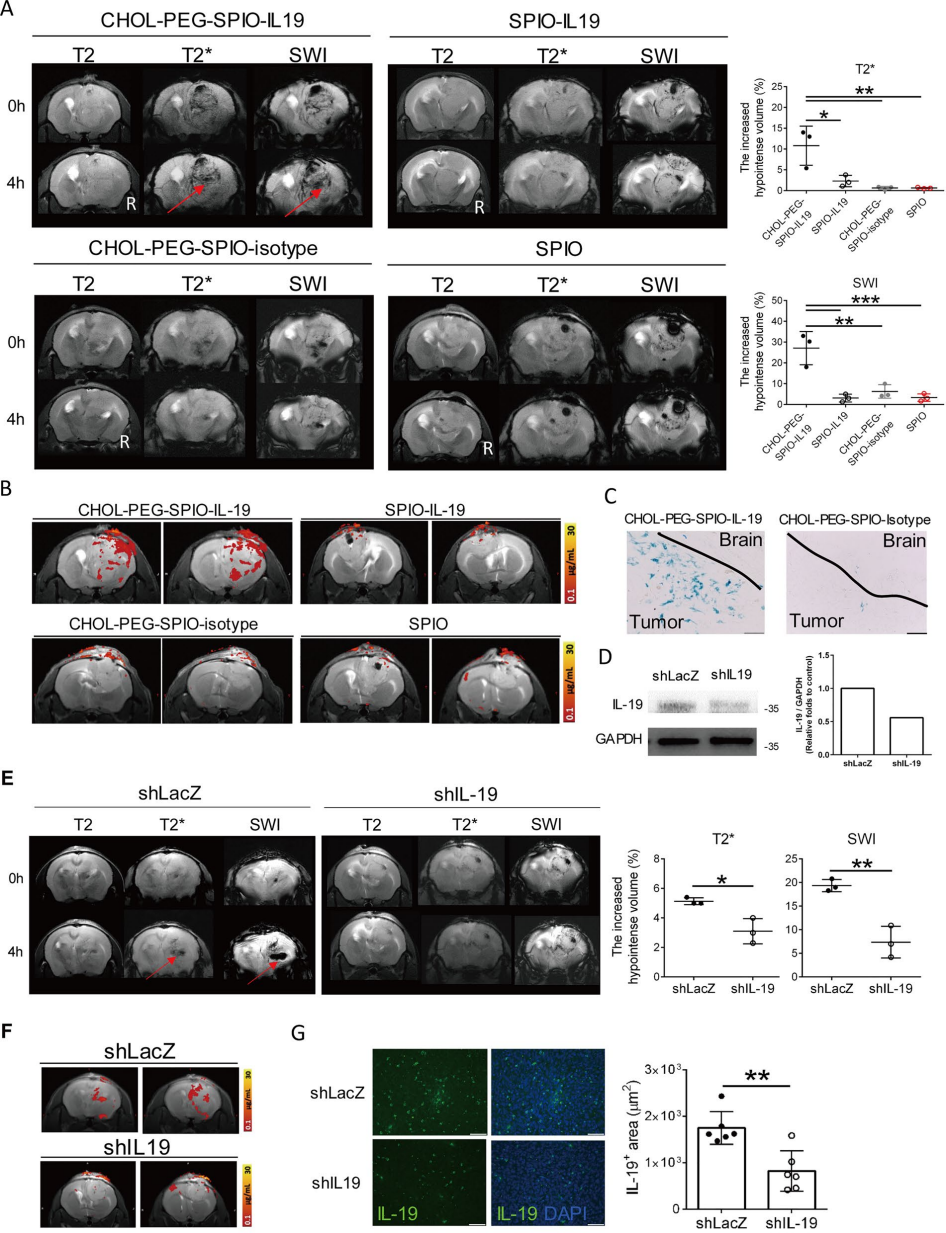
In vivo MRI detection of IL-19 expression in human GSC-derived tumor-bearing mice. **A** In vivo MRI detection of CHOL-PEG-SPIO-IL-19 nanoparticles in human GSC-derived tumor-bearing mice on tumor inoculation day 13. T2-weighted, T2*-weighted, and SWI images of tumor-bearing mice before (0 h) and 4 h after intravenous injection with CHOL-PEG-SPIO-IL-19, SPIO-IL-19, CHOL-PEG-SPIO-isotype, and SPIO nanoparticles (50 μg/mouse) are shown. Red arrows indicate the increased hypointense areas in the tumor. The letter “R” indicates the right side of the brain. The increased percentage of the hypointense volume in the tumor after nanoparticle injection was quantified. n = 3. **p < 0.01, ***p < 0.005 **B** Representative two consecutive T2*-weighted MRI images from tumor-bearing mice after injection with CHOL-PEG-SPIO-IL-19, SPIO-IL-19, CHOL-PEG-SPIO-isotype, and SPIO nanoparticles (50 μg/mouse). n = 3. **C** Detection of iron in CHOL-PEG-SPIO-IL-19 and CHOL-PEG-SPIO-isotype-treated tumor-bearing mice using a Prussian blue assay. Blue staining indicates the presence of iron in the tissues. n = 3. Scale bar: 50 μM. **D** Immunoblot analysis of IL-19 expression in human GSCs following lentiviral transduction with LacZ shRNA (shLacZ) or IL-19-targeting shRNA. **E** In vivo MRI detection of CHOL-PEG-SPIO-IL-19 nanoparticles in human shLacZ and shIL-19 GSC-derived tumor-bearing mice on tumor inoculation day 13. T2-weighted, T2*-weighted, and SWI images of tumor-bearing mice before (0 h) and 4 h after intravenous injection with CHOL-PEG-SPIO-IL-19 (50 μg/mouse) are shown. Red arrows indicate the increased hypointense areas in the tumor. The increased percentage of the hypointense volume in the whole tumor after CHOL-PEG-SPIO-IL-19 nanoparticle injection was quantified. *p < 0.05, **p < 0.01. **F** Representative two T2*-weighted MRI consecutive images from shLacZ and shIL-19 GSC-derived tumor-bearing mice after injection with CHOL-PEG-SPIO-IL-19 nanoparticles (50 μg/mouse). n = 3. **G** Immunofluorescent staining of the IL-19 in shLacZ and shIL-19 GSC-derived tumor tissue. IL-19^+^ areas in shLacZ and shIL-19 tumor were quantified. **p < 0.01 Scale bar: 50 μM

To assess IL-19 expression in human GBM in vivo, we intravenously administered CHOL-PEG-SPIO-IL19 nanoparticles to the tumor-bearing mice. Magnetic resonance imaging (MRI) was performed using T2-weighted, T2*-weighted, and susceptibility-weighted imaging (SWI) sequences at baseline (0 h) and 4 h post-injection of the nanoparticles. The analysis revealed an increase in the percentage of hypointense volume in the tumor following CHOL-PEG-SPIO-IL19 injection compared to baseline control groups. This increase was significantly greater than that observed in other control groups, including SPIO-IL19 (SPIO without CHOL modification) and CHOL-PEG-SPIO-isotype control antibody (CHOL-PEG-SPIO-isotype) (Fig. 8A).

To further quantify the binding of CHOL-PEG-SPIO-IL19 in brain tumors, nanoparticle signals were quantified using T2* mapping before and after nanoparticle injection. ΔR2* analysis confirmed that CHOL-PEG-SPIO-IL19 was present in the tumor region of tumor-bearing mice, whereas less signal was observed in mice treated with SPIO-IL19, CHOL-PEG-SPIO-isotype, or SPIO, as seen in the T2* concentration map (Fig. 8B). These findings suggest that SPIO modification with CHOL-PEG polymers significantly improved the ability of CHOL-PEG-SPIO-IL19 to detect IL-19 expression in brain tumors by in vivo MRI. To confirm the presence of targeted CHOL-PEG-SPIO-IL19 in GBM tissue, Prussian blue staining was performed to detect Fe in tumors from GBM-bearing mice after CHOL-PEG-SPIO-IL19 or CHOL-PEG-SPIO-isotype nanoparticle injection. Prussian blue staining revealed blue signals in the tumor regions of CHOL-PEG-SPIO-IL19-treated mice but not in CHOL-PEG-SPIO-isotype-treated mice (Fig. 8B), indicating the presence of iron oxide nanoparticles in tumor tissue after CHOL-PEG-SPIO-IL19 injection (Fig. 8C).

We next determined whether CHOL-PEG-SPIO-IL19 could be used to distinguish IL-19 expression levels in human GBM tumors in vivo. To this end, we knocked down IL-19 expression in human GSC cells via lentiviral transduction. The results demonstrated that shIL-19 lentiviral transduction reduced IL-19 expression in GSC cells compared to shLacZ-transduced GSC cells (Fig. 8D). Next, we assessed the targeting efficiency of CHOL-PEG-SPIO-IL19 in shIL-19 and shLacZ tumor-bearing mice. After the injection of CHOL-PEG-SPIO-IL19, the increased percentage of hypointense volume in the shIL-19 group was significantly lower than that in the shLacZ tumor-bearing mice (Fig. 8E). In line with this observation, the signal intensity of CHOL-PEG-SPIO-IL19 in shIL-19 tumor-bearing mice was significantly lower than in the shLacZ control group, as demonstrated by the T2* concentration map (Fig. 8F). Consistent with these findings, we confirmed that IL-19^+^ areas were smaller in shIL-19-derived tumors compared to shLacZ-derived tumors in CHOL-PEG-SPIO-IL19 treated mice (Fig. 8G). These results suggest that CHOL-PEG polymers markedly enhance the targeting efficiency of SPIO in brain tumors, enabling CHOL-PEG-SPIO-IL19 to effectively quantify IL-19 expression levels in human GBM tumors via in vivo MRI.

## Discussion

IL-19, an interleukin with known roles in inflammation and immune modulation, emerges as a promising target in GBM therapy. Unlike Arg1 and STAT3 inhibitors, which generally aim to suppress specific immune-suppressive pathways [26, 49], IL-19 antibody therapy directly modulates the tumor microenvironment by addressing aberrant cytokine signaling. By neutralizing IL-19, this therapy can potentially reprogram the immune system to enhance anti-tumor responses, counteracting the immunosuppressive milieu commonly observed in GBM. Through large-scale genome database computing, this study discovered that high *Il-19* expression level was significantly associated with poor survival in patients with GBM and immunosuppressed responses in the peritumoral region. We demonstrated that blocking IL-19 significantly suppressed tumor progression in both TMZ-sensitive and TMZ-resistant GBM cells. Mechanistically, blocking IL-19 enhanced the T cell subsets activation and disrupted immunosuppressive responses of macrophages by disturbing the expression of immunosuppressive genes (*Arg1*, *CD274*, and *S100a4*). Silencing IL-19 in M2-like macrophages lose its immunosuppressive ability on CD8 T cell activation. For the intrinsic IL-19 role in tumor cells, IL-19 is a pro-tumor cytokine that promotes GBM cell migration and invasion through the AKT/β-catenin/WISP1 pathway. Finally, we demonstrated that cholesterol-PEG polymer can improve the targeting efficiency in GBM when antibody is conjugated with SPIO nanoparticles, and prove feasibility of IL-19 is a diagnostic target in GBM. By utilizing these nanoparticles, we were able to non-invasively quantify IL-19 expression levels in vivo, which could help stratify patients likely to benefit from IL-19-targeted therapies. In summary, our study demonstrate that IL-19 is a potential theranostic target to ablate immune suppression and tumor invasion in GBM.

The STAT3 signaling pathway is critical in regulating immune responses and promoting tumor growth. While STAT3 inhibitors have shown promise, they are often associated with significant toxicity and challenges in the poor cell penetrance, lower bioavailability, and bad targeted delivery efficiency [26]. IL-19 antibody therapy offers a more focused approach by specifically targeting the IL-19-mediated signaling axis without broadly affecting STAT3 pathways. This specificity may mitigate some of the adverse effects linked to systemic STAT3 inhibition while still addressing the underlying immune dysregulation. By inhibiting IL-19, our approach aims to shift the immune landscape towards a more anti-tumorigenic state, potentially enhancing the efficacy of concurrent therapies such as checkpoint inhibitors or cytotoxic agents. This synergistic potential represents a significant advantage of IL-19 antibody therapy, as it can be combined with existing TMZ treatment to improve overall therapeutic outcomes.

TAMs are characterized by their plasticity, heterogeneity, and immunosuppressive activity. TMZ-resistant GBM promotes infiltration of M2 macrophages, resulting in an immunosuppressive environment [7]. Manipulating the plasticity of TAMs by reprogramming M2 macrophage differentiation and inhibiting their immunosuppressive function is a promising strategy for improving the effectiveness of immunotherapy. In this study, our scRNA-seq analysis demonstrated that GBM had a higher M2 macrophage content when they acquired TMZ resistance. Blockade IL-19 exhausted M2 macrophage effector function by inhibiting *Arg1* expression in the microenvironment of TMZ-sensitive GBM. Silencing IL-19 significantly down-regulate Arg1 expression and lower immunosuppressive activity of M2-like macrophage on CD8 T cells activation. Since Arg1 expression is controlled by STAT3 [61], we suggest that IL-19 positively regulates Arg1 expression in M2 macrophage via IL-19/STAT3 axis. However, this blocking IL-19 did not affect Arg1 expression in M2-like macrophage subset (MC04) in TMZ-resistant GBM tumor (data not shown). These results suggest that M2 macrophage *Arg1* expression in TMZ-resistant GBM tumor is regulated independently of IL-19 or involves other compensatory mechanisms. Although this blocking effect did not affect Arg1 expression in TMZ-resistant GBM tumor, blocking *IL-19* still can downregulate the expression of STAT3-associated immunosuppressive genes (*CD274* and *S100A4*) in both TMZ-sensitive and TMZ-resistant GBM tumor. Taken together, blocking IL-19 could disturb the canonical *IL-19*/STAT3 pathway and weaken immunosuppressive tumor microenvironment in GBM.

Invasive TMZ-resistant GBM cells evade surgery and focal therapies, posing a major obstacle to curative treatment. Overactivation of STAT3, AKT and WISP1 is associated with TMZ desensitization, resistance, and invasion in GBM [28, 31, 58, 59]. WISP1 activates the AKT signaling pathway in M2 macrophages and GBM cells, promoting their survival and invasion, respectively [59]. However, the critical factor controlling WISP1 expression in GBM remains unknown. In addition to the role of the WISP1/AKT axis in M2 macrophages within the GBM tumor microenvironment, this study identifies IL-19 as a positive regulator of the AKT/WISP1 axis in TMZ-resistant GBM invasion. The study shows that IL-19-triggered AKT phosphorylation can activate WISP1-mediated invasive activity in TMZ-resistant GBM. Although IL-19 triggers STAT3 activation, silencing IL-19 did not promote resensitization of IL-19KO TMZ-R cells to TMZ treatment (data not shown). In summary, targeting IL-19 mitigates the invasive activity of TMZ-resistant GBM cells by disrupting the IL-19/WISP1 signaling pathway.

The heterogeneity of the GBM tumor microenvironment in different subtypes of GBM, the limitations of immunohistochemistry, and the dynamic changes in the target gene expression profile during treatment are current challenges in immunotherapy for GBM [63]. How to specifically select proper patients for suitable target therapy could greatly enhance the therapeutic efficacy. Our study demonstrates the successful development of a novel CHOL-PEG-modified SPIO nanoparticle system conjugated with IL-19 antibodies could overcome this limitation and improves the targeting efficiency. The CHOL-PEG coating significantly enhances the nanoparticle’s ability to target GBM tumors in the brains of animals. This dual-modification approach potentially enhances the stability of the SPIO nanoparticles and reduces the rate of macrophage clearance, ensuring more nanoparticles reach the tumor site. Furthermore, the conjugation of IL-19 antibodies to the nanoparticles enables precise targeting of IL-19-expressing tumor cells. By utilizing MRI, we demonstrated that CHOL-PEG-SPIO-IL19 can non-invasively quantify IL-19 expression levels in vivo, offering a promising theranostic tool that integrates both diagnostic and therapeutic capabilities. This nanoparticle system also holds potential for patient stratification, identifying those who may benefit from IL-19-targeted therapies. These factors position this nanoparticle system as a powerful candidate for future clinical applications, particularly in improving the accuracy of GBM diagnostics and enhancing the efficacy of targeted immunotherapy.

Current immunotherapies predominantly target the effector arm of the immune system, focusing on enhancing T-cell responses through approaches such as immune checkpoint blockade (e.g., PD-L1 antibodies), activation of adaptive immune responses via tumor vaccines, or the use of engineered T cells (e.g., CAR-T therapy) [42]. However, these approaches often yield suboptimal results in treating GBM potentially due to the highly immunosuppressive tumor microenvironment [40]. Given the limited efficacy of single-agent immunotherapies in such a challenging environment, combination therapies integrating diverse immune-based approaches represent a promising strategy to overcome immunotherapy resistance. Our findings reveal that IL-19 is a critical regulator of Arg1-mediated T-cell suppression in GBM, implicating its pivotal role in maintaining the immunosuppressive TME. This suggests that therapeutic strategies combining IL-19 blockade with immune checkpoint inhibitors or adoptive T-cell therapies could synergistically disrupt immunosuppressive mechanisms within the tumor microenvironment. Such combinatorial approaches have the potential to enhance T-cell activation and counter immune evasion, thereby improving therapeutic efficacy in GBM patients. Thus, IL-19-targeted therapy, combined with a theranostic approach, could serve as a valuable tool to effectively synergize with or enhance the efficacy of emerging immune checkpoint inhibitors and T-cell therapies.

This study has several limitations that warrant consideration. While the findings demonstrate promising results in preclinical models, their translation to human patients involves additional complexities, including variations in disease pathophysiology, treatment protocols, and patient-specific characteristics. The scalability of nanoparticle-based therapies poses challenges, particularly regarding production costs, the standardization of manufacturing processes, and regulatory compliance [8, 17]. Although this study primarily investigated the theranostic efficacy of CHOL-PEG-modified SPIO nanoparticles, further studies are required to comprehensively evaluate the potential risks associated with nanoparticle-based therapies, such as toxicity, immune responses, and clearance mechanisms, as well as their biocompatibility and long-term effects. Strategies to address these safety concerns should include optimizing nanoparticle size and surface charge, as both factors have been shown to significantly influence toxicity and biodistribution [65]. Moreover, future clinical trials are essential to assess the safety, efficacy, and long-term outcomes of this therapy across diverse patient populations, addressing the variability in patient responses under clinical settings. Additionally, further studies using additional patient-derived samples are necessary to determine the IL-19 protein expression level and to assess whether IL-19 antibodies can effectively inhibit tumor progression.

## Conclusion

Joint targeting of the immunosuppressive and invasive characteristics of GBM cells can improve the immunotherapy efficacy. IL-19 antibody therapy could be combined with TMZ treatment to improve overall therapeutic outcomes. Its potential to directly target aberrant cytokine signaling, coupled with a more selective impact on the immunosuppressive M2 macrophage and tumor invasiveness in TMZ-resistant GBM microenvironment. The development of IL-19-targeted nanoparticles further highlights the potential of IL-19 as a theranostic agent in GBM treatment. While IL-19 shows promise as a theranostic target, our study was conducted in preclinical models, and further validation in clinical settings is essential. The challenges related to the translational potential of nanoparticle-based therapies, the complexity of GBM heterogeneity, and the variability in patient responses, are critical to consider as we move toward clinical applications. To evaluate the safety and efficacy of IL-19-targeted therapies in animal models, optimizing nanoparticle delivery systems, overcoming scalability challenges, and generating humanized IL-19 antibody would bring this therapy closer to clinical application.

## Supplementary Information

Additional file 1.

Additional file 2.

## Data Availability

The datasets used and analyzed during the current study are available from the corresponding author upon reasonable request.

## References

[1] Abdelfattah N, Kumar P, Wang C, Leu JS, Flynn WF, Gao R, Baskin DS, Pichumani K, Ijare OB, Wood SL, Powell SZ, Haviland DL, Parker Kerrigan BC, Lang FF, Prabhu SS, Huntoon KM, Jiang W, Kim BYS, George J, Yun K. Single-cell analysis of human glioma and immune cells identifies S100A4 as an immunotherapy target. Nat Commun. 2022;13(1):767. 35140215 10.1038/s41467-022-28372-y PMC8828877

[2] Abedalthagafi M, Barakeh D, Foshay KM. Immunogenetics of glioblastoma: the future of personalized patient management. NPJ Precis Oncol. 2018;2:27. 30534602 10.1038/s41698-018-0070-1 PMC6279755

[3] Azuma YT, Matsuo Y, Kuwamura M, Yancopoulos GD, Valenzuela DM, Murphy AJ, Nakajima H, Karow M, Takeuchi T. Interleukin-19 protects mice from innate-mediated colonic inflammation. Inflamm Bowel Dis. 2010;16(6):1017–28. 19834971 10.1002/ibd.21151

[4] Bergmann N, Delbridge C, Gempt J, Feuchtinger A, Walch A, Schirmer L, Bunk W, Aschenbrenner T, Liesche-Starnecker F, Schlegel J. The intratumoral heterogeneity reflects the intertumoral subtypes of glioblastoma multiforme: a regional immunohistochemistry analysis. Front Oncol. 2020;10:494. 32391260 10.3389/fonc.2020.00494 PMC7193089

[5] Cantini G, Pisati F, Mastropietro A, Frattini V, Iwakura Y, Finocchiaro G, Pellegatta S. A critical role for regulatory T cells in driving cytokine profiles of Th17 cells and their modulation of glioma microenvironment. Cancer Immunol Immunother. 2011;60(12):1739–50. 21779877 10.1007/s00262-011-1069-4 PMC11028703

[6] Ceccarelli M, Barthel FP, Malta TM, Sabedot TS, Salama SR, Murray BA, Morozova O, Newton Y, Radenbaugh A, Pagnotta SM, Anjum S, Wang J, Manyam G, Zoppoli P, Ling S, Rao AA, Grifford M, Cherniack AD, Zhang H, Poisson L, Carlotti CG Jr, Tirapelli DP, Rao A, Mikkelsen T, Lau CC, Yung WK, Rabadan R, Huse J, Brat DJ, Lehman NL, Barnholtz-Sloan JS, Zheng S, Hess K, Rao G, Meyerson M, Beroukhim R, Cooper L, Akbani R, Wrensch M, Haussler D, Aldape KD, Laird PW, Gutmann DH, Network TR, Noushmehr H, Iavarone A, Verhaak RG. Molecular profiling reveals biologically discrete subsets and pathways of progression in diffuse glioma. Cell. 2016;164(3):550–63. 26824661 10.1016/j.cell.2015.12.028 PMC4754110

[7] Di Ianni N, Maffezzini M, Eoli M, Pellegatta S. Revisiting the immunological aspects of temozolomide considering the genetic landscape and the immune microenvironment composition of glioblastoma. Front Oncol. 2021;11: 747690. 34646780 10.3389/fonc.2021.747690 PMC8503270

[8] Dordevic S, Gonzalez MM, Conejos-Sanchez I, Carreira B, Pozzi S, Acurcio RC, Satchi-Fainaro R, Florindo HF, Vicent MJ. Current hurdles to the translation of nanomedicines from bench to the clinic. Drug Deliv Transl Res. 2022;12(3):500–25. 34302274 10.1007/s13346-021-01024-2 PMC8300981

[9] Doucette T, Rao G, Rao A, Shen L, Aldape K, Wei J, Dziurzynski K, Gilbert M, Heimberger AB. Immune heterogeneity of glioblastoma subtypes: extrapolation from the cancer genome atlas. Cancer Immunol Res. 2013;1(2):112–22. 24409449 10.1158/2326-6066.CIR-13-0028 PMC3881271

[10] El Andaloussi A, Lesniak MS. An increase in CD4+CD25+FOXP3+ regulatory T cells in tumor-infiltrating lymphocytes of human glioblastoma multiforme. Neuro Oncol. 2006;8(3):234–43. 16723631 10.1215/15228517-2006-006 PMC1871953

[11] Fang D, Hawke D, Zheng Y, Xia Y, Meisenhelder J, Nika H, Mills GB, Kobayashi R, Hunter T, Lu Z. Phosphorylation of beta-catenin by AKT promotes beta-catenin transcriptional activity. J Biol Chem. 2007;282(15):11221–9. 17287208 10.1074/jbc.M611871200 PMC1850976

[12] Fridman WH, Pages F, Sautes-Fridman C, Galon J. The immune contexture in human tumours: impact on clinical outcome. Nat Rev Cancer. 2012;12(4):298–306. 22419253 10.1038/nrc3245

[13] Gabunia K, Autieri MV. Interleukin-19 can enhance angiogenesis by Macrophage Polarization. Macrophage (Houst). 2015;2(1): e562. 26029742 10.14800/macrophage.562 PMC4447484

[14] Gadani SP, Cronk JC, Norris GT, Kipnis J. IL-4 in the brain: a cytokine to remember. J Immunol. 2012;189(9):4213–9. 23087426 10.4049/jimmunol.1202246 PMC3481177

[15] Gallagher G. Interleukin-19: multiple roles in immune regulation and disease. Cytokine Growth Factor Rev. 2010;21(5):345–52. 20889366 10.1016/j.cytogfr.2010.08.005

[16] Gao H, Qian J, Cao S, Yang Z, Pang Z, Pan S, Fan L, Xi Z, Jiang X, Zhang Q. Precise glioma targeting of and penetration by aptamer and peptide dual-functioned nanoparticles. Biomaterials. 2012;33(20):5115–23. 22484043 10.1016/j.biomaterials.2012.03.058

[17] Gavas S, Quazi S, Karpinski TM. Nanoparticles for cancer therapy: current progress and challenges. Nanoscale Res Lett. 2021;16(1):173. 34866166 10.1186/s11671-021-03628-6 PMC8645667

[18] Grzywa TM, Sosnowska A, Matryba P, Rydzynska Z, Jasinski M, Nowis D, Golab J. Myeloid cell-derived arginase in cancer immune response. Front Immunol. 2020;11:938. 32499785 10.3389/fimmu.2020.00938 PMC7242730

[19] Hao C, Chen G, Zhao H, Li Y, Chen J, Zhang H, Li S, Zhao Y, Chen F, Li W, Jiang WG. PD-L1 expression in glioblastoma, the clinical and prognostic significance: a systematic literature review and meta-analysis. Front Oncol. 2020;10:1015. 32670884 10.3389/fonc.2020.01015 PMC7326811

[20] Heynckes S, Gaebelein A, Haaker G, Grauvogel J, Franco P, Mader I, Carro MS, Prinz M, Delev D, Schnell O, Heiland DH. Expression differences of programmed death ligand 1 in de-novo and recurrent glioblastoma multiforme. Oncotarget. 2017;8(43):74170–7. 29088776 10.18632/oncotarget.18819 PMC5650331

[21] Horiuchi H, Parajuli B, Komiya H, Ogawa Y, Jin S, Takahashi K, Azuma YT, Tanaka F, Suzumura A, Takeuchi H. Interleukin-19 abrogates experimental autoimmune encephalomyelitis by attenuating antigen-presenting cell activation. Front Immunol. 2021;12: 615898. 33776998 10.3389/fimmu.2021.615898 PMC7990911

[22] Horiuchi H, Parajuli B, Wang Y, Azuma YT, Mizuno T, Takeuchi H, Suzumura A. Interleukin-19 acts as a negative autocrine regulator of activated microglia. PLoS ONE. 2015;10(3): e0118640. 25794104 10.1371/journal.pone.0118640 PMC4368203

[23] Hsing CH, Cheng HC, Hsu YH, Chan CH, Yeh CH, Li CF, Chang MS. Upregulated IL-19 in breast cancer promotes tumor progression and affects clinical outcome. Clin Cancer Res. 2012;18(3):713–25. 22186257 10.1158/1078-0432.CCR-11-1532

[24] Hsing CH, Kwok FA, Cheng HC, Li CF, Chang MS. Inhibiting interleukin-19 activity ameliorates esophageal squamous cell carcinoma progression. PLoS ONE. 2013;8(10): e75254. 24130695 10.1371/journal.pone.0075254 PMC3793994

[25] Hu X, Yang F, Liao Y, Li L, Zhang L. Cholesterol-PEG comodified poly (N-butyl) cyanoacrylate nanoparticles for brain delivery: in vitro and in vivo evaluations. Drug Deliv. 2017;24(1):121–32. 28156159 10.1080/10717544.2016.1233590 PMC8241168

[26] Hu Y, Dong Z, Liu K. Unraveling the complexity of STAT3 in cancer: molecular understanding and drug discovery. J Exp Clin Cancer Res. 2024;43(1):23. 38245798 10.1186/s13046-024-02949-5 PMC10799433

[27] Itakura H, Achrol AS, Mitchell LA, Loya JJ, Liu T, Westbroek EM, Feroze AH, Rodriguez S, Echegaray S, Azad TD, Yeom KW, Napel S, Rubin DL, Chang SD, Harsh GRT, Gevaert O. Magnetic resonance image features identify glioblastoma phenotypic subtypes with distinct molecular pathway activities. Sci Transl Med. 2015;7(303):303ra138. 26333934 10.1126/scitranslmed.aaa7582 PMC4666025

[28] Jing D, Zhang Q, Yu H, Zhao Y, Shen L. Identification of WISP1 as a novel oncogene in glioblastoma. Int J Oncol. 2017;51(4):1261–70. 28902353 10.3892/ijo.2017.4119

[29] Kang I, Kim Y, Lee HK. gammadelta T cells as a potential therapeutic agent for glioblastoma. Front Immunol. 2023;14:1273986. 37928546 10.3389/fimmu.2023.1273986 PMC10623054

[30] Kindy MS, Yu J, Zhu H, Smith MT, Gattoni-Celli S. A therapeutic cancer vaccine against GL261 murine glioma. J Transl Med. 2016;14:1. 26727970 10.1186/s12967-015-0757-9 PMC4700644

[31] Kohsaka S, Wang L, Yachi K, Mahabir R, Narita T, Itoh T, Tanino M, Kimura T, Nishihara H, Tanaka S. STAT3 inhibition overcomes temozolomide resistance in glioblastoma by downregulating MGMT expression. Mol Cancer Ther. 2012;11(6):1289–99. 22532597 10.1158/1535-7163.MCT-11-0801

[32] Koul D, Shen R, Bergh S, Sheng X, Shishodia S, Lafortune TA, Lu Y, de Groot JF, Mills GB, Yung WK. Inhibition of Akt survival pathway by a small-molecule inhibitor in human glioblastoma. Mol Cancer Ther. 2006;5(3):637–44. 16546978 10.1158/1535-7163.MCT-05-0453

[33] Kumar NP, Moideen K, Banurekha VV, Nair D, Babu S. Modulation of Th1/Tc1 and Th17/Tc17 responses in pulmonary tuberculosis by IL-20 subfamily of cytokines. Cytokine. 2018;108:190–6. 29684756 10.1016/j.cyto.2018.04.005 PMC5962435

[34] Lee GA, Lin WL, Kuo DP, Li YT, Chang YW, Chen YC, Huang SW, Hsu JBK, Chen CY. Detection of PD-L1 expression in temozolomide-resistant glioblastoma by using PD-L1 antibodies conjugated with lipid-coated superparamagnetic iron oxide. Int J Nanomed. 2021;16:5233–46. 10.2147/IJN.S310464 PMC8336995 34366665

[35] Lee GA, Lin WL, Kuo DP, Li YT, Chang YW, Chen YC, Huang SW, Hsu JB, Chen CY. Detection of PD-L1 expression in temozolomide-resistant glioblastoma by using PD-L1 antibodies conjugated with lipidcoated superparamagnetic iron oxide. Int J Nanomed. 2021;16:5233–46. 10.2147/IJN.S310464 PMC8336995 34366665

[36] Leigh T, Scalia RG, Autieri MV. Resolution of inflammation in immune and nonimmune cells by interleukin-19. Am J Physiol Cell Physiol. 2020;319(3):C457–64. 32667867 10.1152/ajpcell.00247.2020 PMC7509264

[37] Lemee JM, Clavreul A, Menei P. Intratumoral heterogeneity in glioblastoma: don’t forget the peritumoral brain zone. Neuro Oncol. 2015;17(10):1322–32. 26203067 10.1093/neuonc/nov119 PMC4578587

[38] Lewis BW, Patial S, Saini Y. In vitro screening method for characterization of macrophage activation responses. Methods Protoc. 2022;5(5):68. 36136814 10.3390/mps5050068 PMC9498385

[39] Li J, Ye L, Owen S, Weeks HP, Zhang Z, Jiang WG. Emerging role of CCN family proteins in tumorigenesis and cancer metastasis (Review). Int J Mol Med. 2015;36(6):1451–63. 26498181 10.3892/ijmm.2015.2390 PMC4678164

[40] Li Y-R, Halladay T, Yang L. Immune evasion in cell-based immunotherapy: unraveling challenges and novel strategies. J Biomed Sci. 2024;31(1):5. 38217016 10.1186/s12929-024-00998-8 PMC10785504

[41] Liu T, Li Y, Lin K, Yin H, He B, Zheng M, Wang G. Regulation of S100A4 expression via the JAK2-STAT3 pathway in rhomboid-phenotype pulmonary arterial smooth muscle cells exposure to hypoxia. Int J Biochem Cell Biol. 2012;44(8):1337–45. 22561747 10.1016/j.biocel.2012.04.017

[42] Liu Y, Zhou F, Ali H, Lathia JD, Chen P. Immunotherapy for glioblastoma: current state, challenges, and future perspectives. Cell Mol Immunol. 2024;21(12):1354–75. 39406966 10.1038/s41423-024-01226-x PMC11607068

[43] Lynes J, Sanchez V, Dominah G, Nwankwo A, Nduom E. Current options and future directions in immune therapy for glioblastoma. Front Oncol. 2018;8:578. 30568917 10.3389/fonc.2018.00578 PMC6290347

[44] Marzec M, Zhang Q, Goradia A, Raghunath PN, Liu X, Paessler M, Wang HY, Wysocka M, Cheng M, Ruggeri BA, Wasik MA. Oncogenic kinase NPM/ALK induces through STAT3 expression of immunosuppressive protein CD274 (PD-L1, B7–H1). Proc Natl Acad Sci U S A. 2008;105(52):20852–7. 19088198 10.1073/pnas.0810958105 PMC2634900

[45] McIntosh BE, Brown ME, Duffin BM, Maufort JP, Vereide DT, Slukvin II, Thomson JA. Nonirradiated NOD, B6.SCID Il2rgamma-/- Kit(W41/W41) (NBSGW) mice support multilineage engraftment of human hematopoietic cells. Stem Cell Reports. 2015;4(2):171–80. 25601207 10.1016/j.stemcr.2014.12.005 PMC4325197

[46] Mi Y, Guo N, Luan J, Cheng J, Hu Z, Jiang P, Jin W, Gao X. The emerging role of myeloid-derived suppressor cells in the glioma immune suppressive microenvironment. Front Immunol. 2020;11:737. 32391020 10.3389/fimmu.2020.00737 PMC7193311

[47] Nagashima G, Suzuki R, Hokaku H, Takahashi M, Miyo T, Asai J, Nakagawa N, Fujimoto T. Graphic analysis of microscopic tumor cell infiltration, proliferative potential, and vascular endothelial growth factor expression in an autopsy brain with glioblastoma. Surg Neurol. 1999;51(3):292–9. 10086494 10.1016/s0090-3019(98)00056-1

[48] Nduom EK, Wei J, Yaghi NK, Huang N, Kong LY, Gabrusiewicz K, Ling X, Zhou S, Ivan C, Chen JQ, Burks JK, Fuller GN, Calin GA, Conrad CA, Creasy C, Ritthipichai K, Radvanyi L, Heimberger AB. PD-L1 expression and prognostic impact in glioblastoma. Neuro Oncol. 2016;18(2):195–205. 26323609 10.1093/neuonc/nov172 PMC4724183

[49] Niu F, Yu Y, Li Z, Ren Y, Li Z, Ye Q, Liu P, Ji C, Qian L, Xiong Y. Arginase: an emerging and promising therapeutic target for cancer treatment. Biomed Pharmacother. 2022;149: 112840. 35316752 10.1016/j.biopha.2022.112840

[50] Ochocka N, Segit P, Walentynowicz KA, Wojnicki K, Cyranowski S, Swatler J, Mieczkowski J, Kaminska B. Single-cell RNA sequencing reveals functional heterogeneity of glioma-associated brain macrophages. Nat Commun. 2021;12(1):1151. 33608526 10.1038/s41467-021-21407-w PMC7895824

[51] Ostrom QT, Gittleman H, Fulop J, Liu M, Blanda R, Kromer C, Wolinsky Y, Kruchko C, Barnholtz-Sloan JS. CBTRUS statistical report: primary brain and central nervous system tumors diagnosed in the United States in 2008–2012. Neuro Oncol. 2015;17(Suppl 4):iv1–62. 26511214 10.1093/neuonc/nov189 PMC4623240

[52] Paladugu M, Thakur A, Lum LG, Mittal S, Parajuli P. Generation and immunologic functions of Th17 cells in malignant gliomas. Cancer Immunol Immunother. 2013;62(1):75–86. 22752645 10.1007/s00262-012-1312-7 PMC3888106

[53] Pearson JRD, Cuzzubbo S, McArthur S, Durrant LG, Adhikaree J, Tinsley CJ, Pockley AG, McArdle SEB. Immune escape in glioblastoma multiforme and the adaptation of immunotherapies for treatment. Front Immunol. 2020;11: 582106. 33178210 10.3389/fimmu.2020.582106 PMC7594513

[54] Peng XH, Qian X, Mao H, Wang AY, Chen ZG, Nie S, Shin DM. Targeted magnetic iron oxide nanoparticles for tumor imaging and therapy. Int J Nanomedicine. 2008;3(3):311–21. 18990940 10.2147/ijn.s2824 PMC2626938

[55] Petrecca K, Guiot MC, Panet-Raymond V, Souhami L. Failure pattern following complete resection plus radiotherapy and temozolomide is at the resection margin in patients with glioblastoma. J Neurooncol. 2013;111(1):19–23. 23054563 10.1007/s11060-012-0983-4

[56] Rochigneux P, Garcia AJ, Chanez B, Madroszyk A, Olive D, Garon EB. Medical treatment of lung cancer: can immune cells predict the response? A systematic review. Front Immunol. 2020;11:1036. 32670271 10.3389/fimmu.2020.01036 PMC7327092

[57] Sherriff J, Tamangani J, Senthil L, Cruickshank G, Spooner D, Jones B, Brookes C, Sanghera P. Patterns of relapse in glioblastoma multiforme following concomitant chemoradiotherapy with temozolomide. Br J Radiol. 2013;86(1022):20120414. 23385995 10.1259/bjr.20120414 PMC3608050

[58] Singh N, Miner A, Hennis L, Mittal S. Mechanisms of temozolomide resistance in glioblastoma—a comprehensive review. Cancer Drug Resist. 2021;4:17–43. 34337348 10.20517/cdr.2020.79 PMC8319838

[59] Tao W, Chu C, Zhou W, Huang Z, Zhai K, Fang X, Huang Q, Zhang A, Wang X, Yu X, Huang H, Wu Q, Sloan AE, Yu JS, Li X, Stark GR, Rich JN, Bao S. Dual Role of WISP1 in maintaining glioma stem cells and tumor-supportive macrophages in glioblastoma. Nat Commun. 2020;11(1):3015. 32541784 10.1038/s41467-020-16827-z PMC7295765

[60] Tsou YH, Zhang XQ, Zhu H, Syed S, Xu X. Drug delivery to the brain across the blood-brain barrier using nanomaterials. Small. 2017;13(43). 10.1002/smll.201701921 29045030

[61] Vasquez-Dunddel D, Pan F, Zeng Q, Gorbounov M, Albesiano E, Fu J, Blosser RL, Tam AJ, Bruno T, Zhang H, Pardoll D, Kim Y. STAT3 regulates arginase-I in myeloid-derived suppressor cells from cancer patients. J Clin Invest. 2013;123(4):1580–9. 23454751 10.1172/JCI60083 PMC3613901

[62] Vauleon E, Tony A, Hamlat A, Etcheverry A, Chiforeanu DC, Menei P, Mosser J, Quillien V, Aubry M. Immune genes are associated with human glioblastoma pathology and patient survival. BMC Med Genomics. 2012;5:41. 22980038 10.1186/1755-8794-5-41 PMC3507656

[63] Wang X, Guo G, Guan H, Yu Y, Lu J, Yu J. Challenges and potential of PD-1/PD-L1 checkpoint blockade immunotherapy for glioblastoma. J Exp Clin Cancer Res. 2019;38(1):87. 30777100 10.1186/s13046-019-1085-3 PMC6380009

[64] Wang X, Lu J, Guo G, Yu J. Immunotherapy for recurrent glioblastoma: practical insights and challenging prospects. Cell Death Dis. 2021;12(4):299. 33741903 10.1038/s41419-021-03568-0 PMC7979733

[65] Wei H, Hu Y, Wang J, Gao X, Qian X, Tang M. Superparamagnetic iron oxide nanoparticles: cytotoxicity, metabolism, and cellular behavior in biomedicine applications. Int J Nanomed. 2021;16:6097–113. 10.2147/IJN.S321984 PMC8418330 34511908

[66] Wu J. The enhanced permeability and retention (EPR) effect: the significance of the concept and methods to enhance its application. J Pers Med. 2021;11(8):771. 34442415 10.3390/jpm11080771 PMC8402171

[67] Xin H, Jiang X, Gu J, Sha X, Chen L, Law K, Chen Y, Wang X, Jiang Y, Fang X. Angiopep-conjugated poly(ethylene glycol)-co-poly(ε-caprolactone) nanoparticles as dual-targeting drug delivery system for brain glioma. Biomaterials. 2011;32(18):4293–305. 21427009 10.1016/j.biomaterials.2011.02.044

[68] Yamahara T, Numa Y, Oishi T, Kawaguchi T, Seno T, Asai A, Kawamoto K. Morphological and flow cytometric analysis of cell infiltration in glioblastoma: a comparison of autopsy brain and neuroimaging. Brain Tumor Pathol. 2010;27(2):81–7. 21046309 10.1007/s10014-010-0275-7

[69] Zhang J, Bajari R, Andric D, Gerthoffert F, Lepsa A, Nahal-Bose H, Stein LD, Ferretti V. The international cancer genome consortium data portal. Nat Biotechnol. 2019;37(4):367–9. 30877282 10.1038/s41587-019-0055-9

[70] Zhang JF, Tao T, Wang K, Zhang GX, Yan Y, Lin HR, Li Y, Guan MW, Yu JJ, Wang XD. IL-33/ST2 axis promotes glioblastoma cell invasion by accumulating tenascin-C. Sci Rep. 2019;9(1):20276. 31889095 10.1038/s41598-019-56696-1 PMC6937274

[71] Zhong Y, Meng F, Deng C, Zhong Z. Ligand-directed active tumor-targeting polymeric nanoparticles for cancer chemotherapy. Biomacromol. 2014;15(6):1955–69. 10.1021/bm5003009 24798476

[72] Zou J, Tan W, Li B, Wang Z, Li Y, Zeng J, Jiang B, Yoshida S, Zhou Y. Interleukin-19 promotes retinal neovascularization in a mouse model of oxygen-induced retinopathy. Invest Ophthalmol Vis Sci. 2022;63(8):9. 35816041 10.1167/iovs.63.8.9 PMC9284469

