# Supplementary material for: IL-19 as a promising theranostic target to reprogram the glioblastoma immunosuppressive microenvironment
Source: J Biomed Sci. 2025 Mar 8;32:34. doi: 10.1186/s12929-025-01126-w (PMC11889942; doi:10.1186/s12929-025-01126-w)
Supplement: Supplementary file 2 — Additional file 2. [file 12929_2025_1126_MOESM2_ESM.docx]

**Supplementary Figures**

**
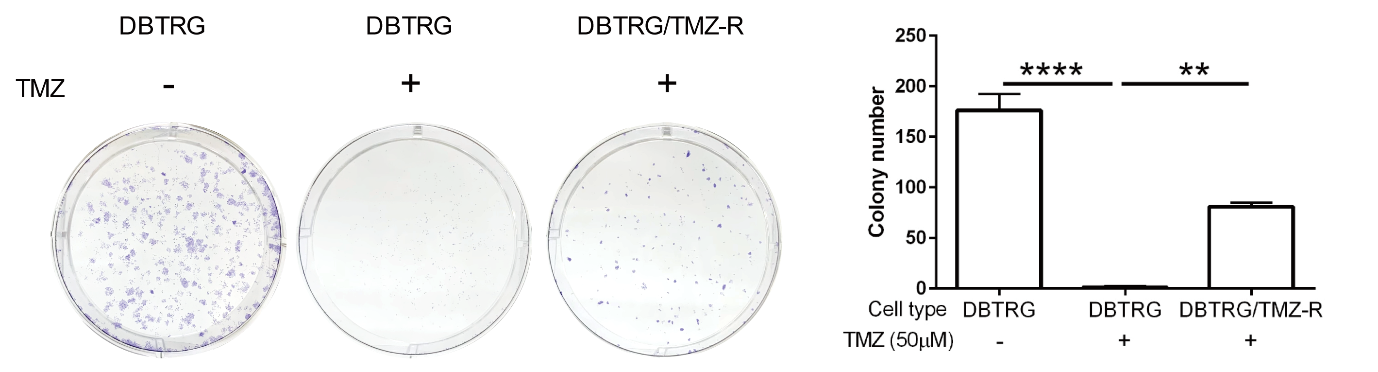
**

**Supplementary Fig. 1 TMZ resistance of DBTRG/TMZ-R cells was confirmed using colony formation assay.** Representative images of DBTRG and DBTRG/TMZ-R cell colonies after 9 days of treatment with 50 μM TMZ are shown. Quantitative results of the colony formation assay are shown as the total number of surviving colonies/well (*p < 0.05; n = 3)

**
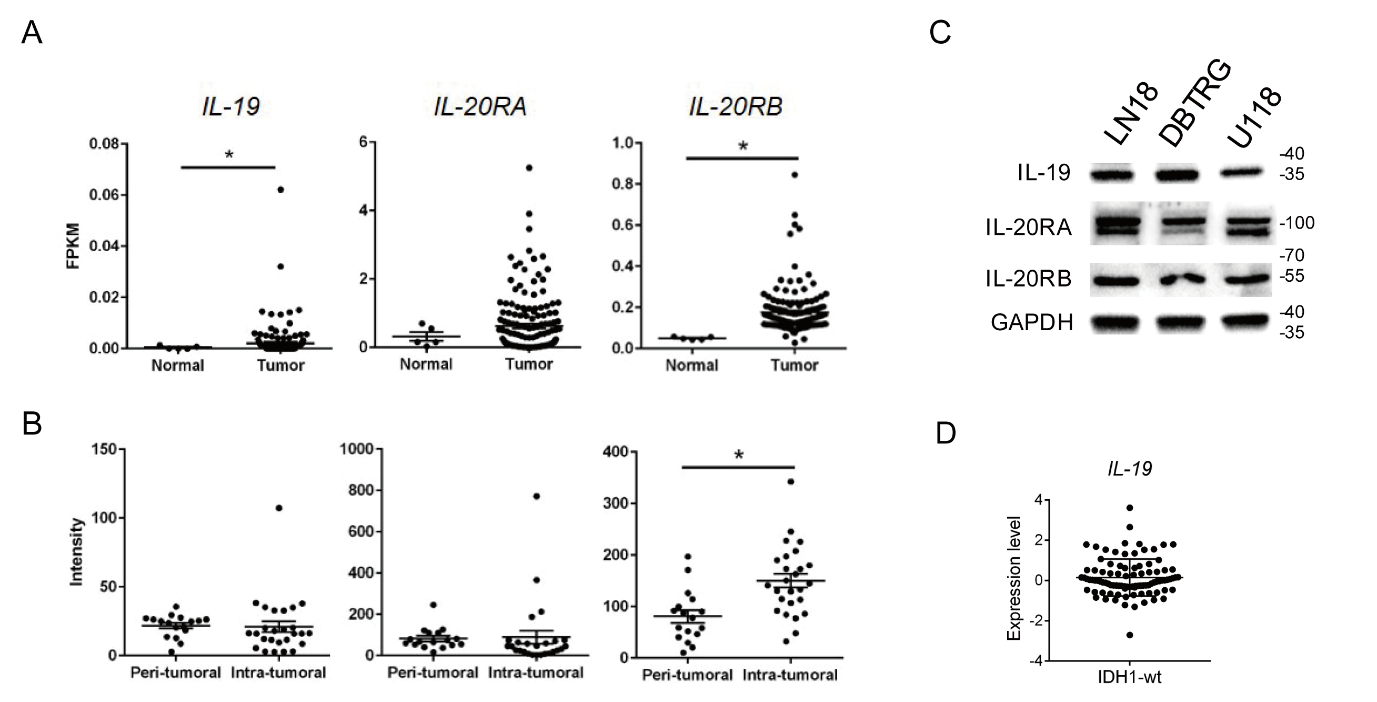
**

**Supplementary Fig. 2** **The expression of IL-19 and its receptors (IL-20RA and IL-20RB) in GBM tumor tissue and cancer cell lines.**

(A) Expression levels of *IL-19*, *IL-20RA*, and *IL-20RB* in the normal brain tissue and tumor tissue of patients with GBM from RNA-seq data. *** *P* < 0.05. (B) Expression levels of *IL-19*, *IL-20RA*, and *IL-20RB* in the peritumoral tissueand intratumoral tissue of patients with GBM from microarray data in the Taiwan GBM study cohort. *** *P* < 0.05. (C) Expression levels of IL-19, IL-20RA, and IL-20RB in GBM cell lines (LN18, DBTRG, U118) was analyzed by Western blot. (D) IL-19 expression level in tumor tissue of patients with IDH1-wt GBM from CGGA RNA microarray data. n=94.


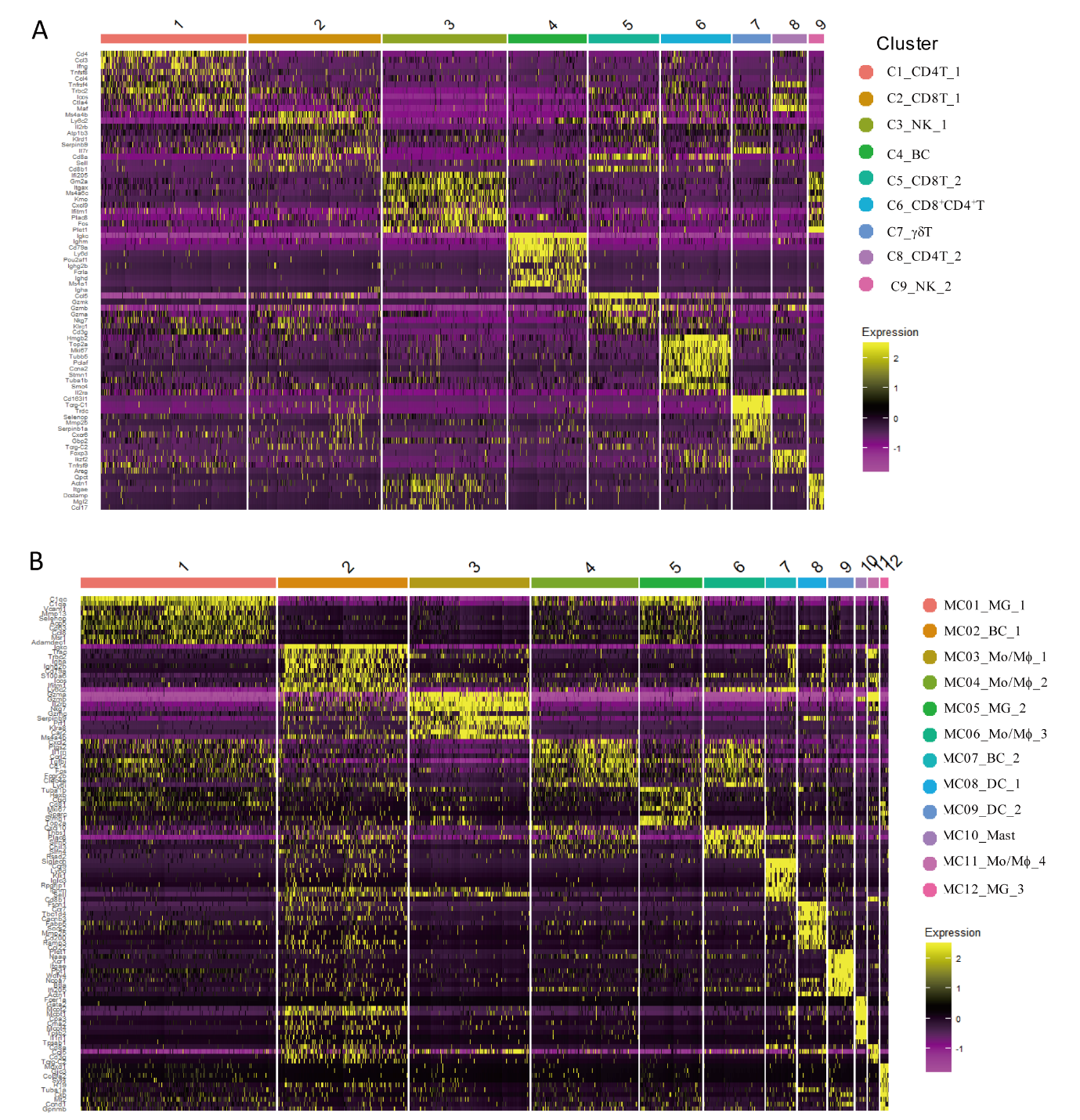


**Supplementary Fig. 3** The top differentially expressed genes profile of these lymphoid and myeloid cells clusters. (A) DEGs are listed in nine lymphoid clusters (C1-C9) and (B) 12 clusters (MC01-MC12, includes 10 myeloid clusters and 2 B cell clusters).


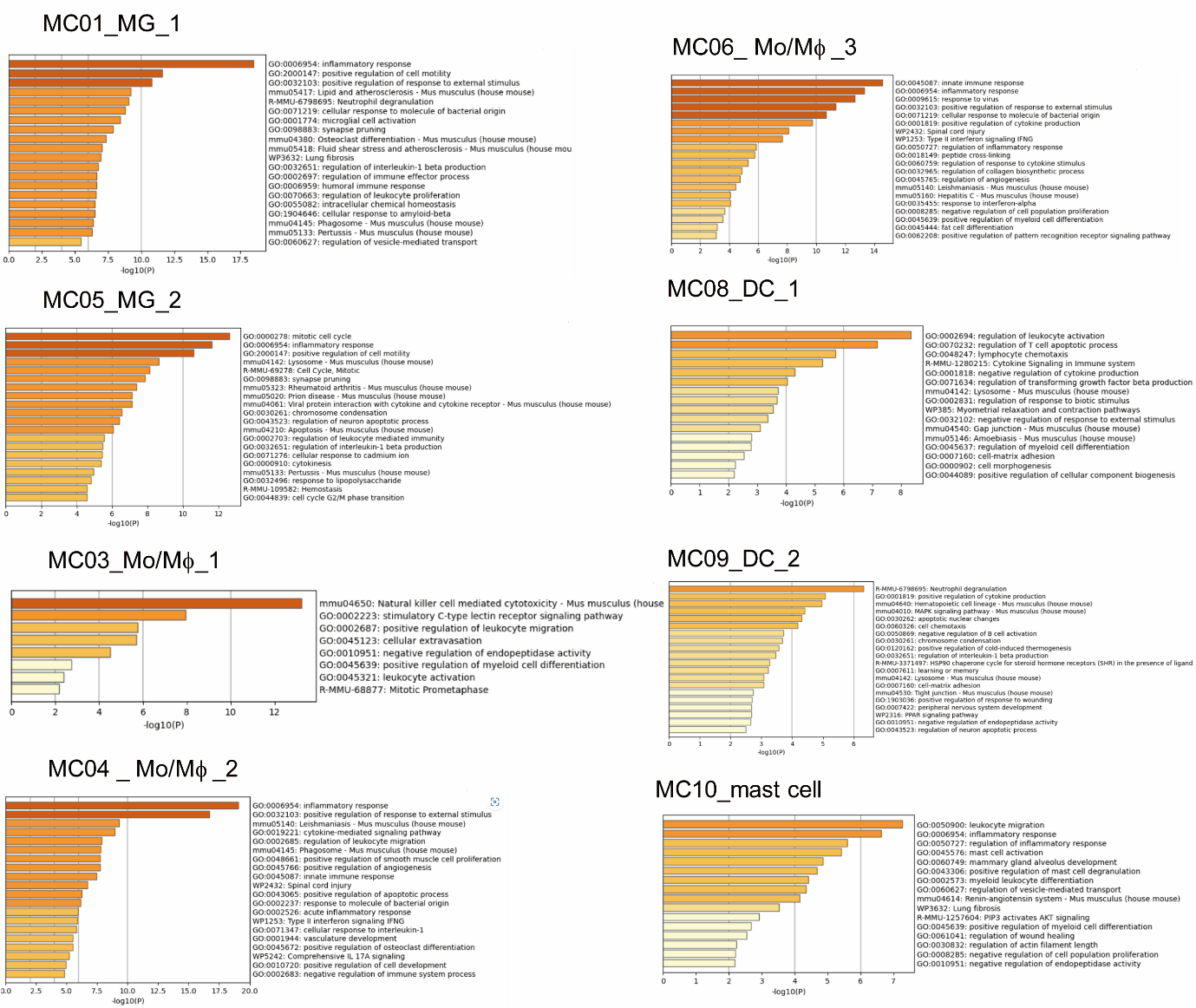


**Supplementary Fig. 4.** **The GO annotation of immune clusters in brain-infiltrating myeloid cells.**

The top 20 GO terms in myeloid subpopulations in the tumor. Upregulated DEGs in two microglia clusters (MC01, MC05), monocyte/macrophage clusters (MC03, MC04, MC06), DC clusters (MC08, MC09), mast cell (MC10) were used for GO annotation enrichment analysis by metascape.


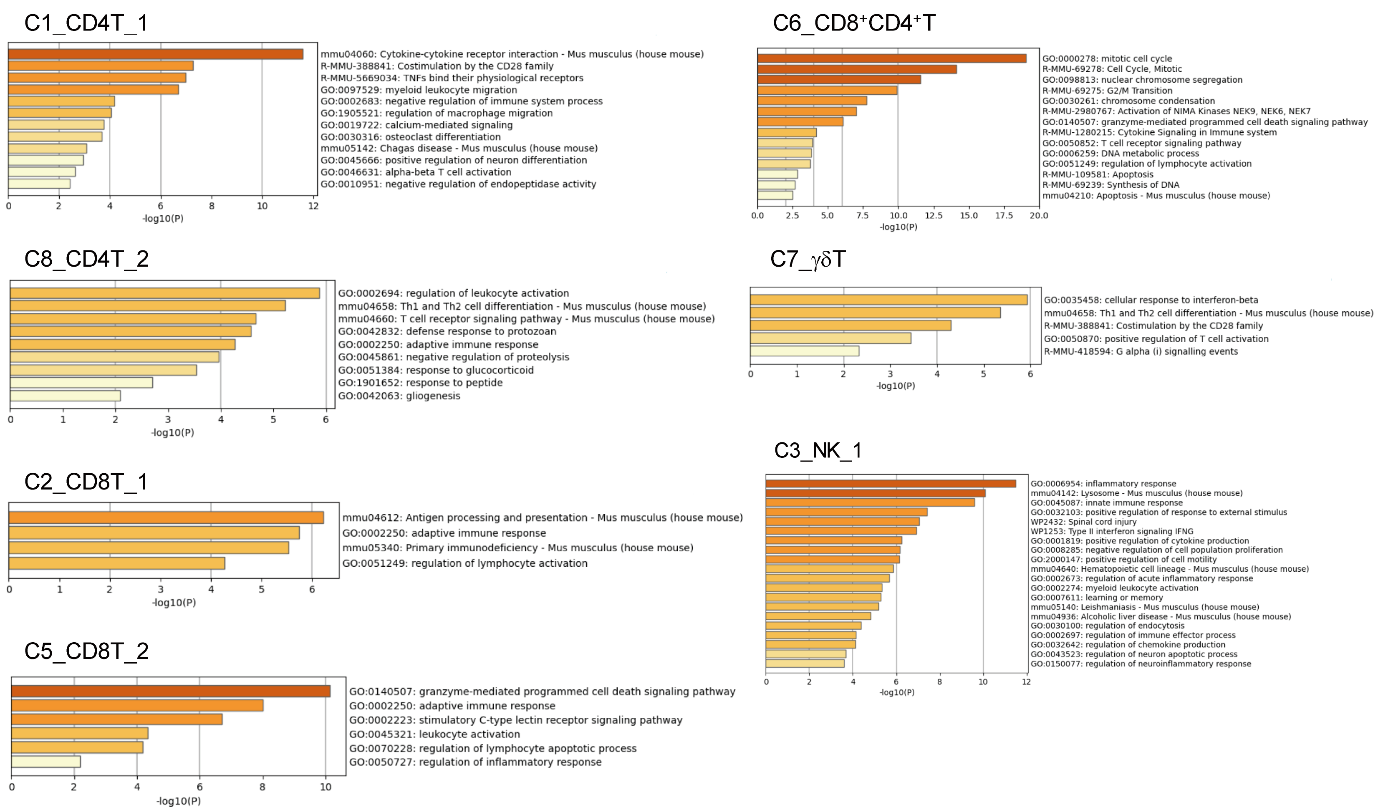


**Supplementary Fig. 5.** **The GO annotation of immune clusters in brain-infiltrating lymphocytes.**

The top 20 GO terms in lymphoid subpopulations in the tumor-infiltrating leukocytes. Upregulated DEGs in two CD4 T cell clusters (C1, C8), two CD8 T cell clusters (C2, C5), one CD8^+^CD4^+^ T cell cluster (C6), and one γδ T cell cluster (C7), DC clusters (C4, C9), T and NK cell cluster (C6), and T and B cell cluster (C10) were used for GO annotation enrichment analysis by metascape.


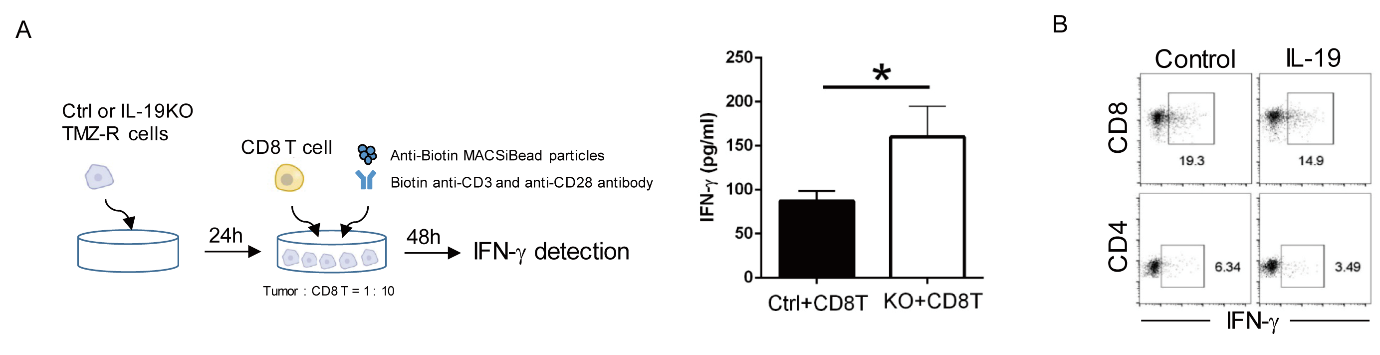
**Supplementary Fig. 6. IL-19 effects on T cells activation.**

(A) Effects of tumor-derived *IL-19* on CD8^+^ TC activation. Ctrl and IL-19KO GL261/TMZ-R cells were cocultured with CD8^+^ TCs for 48 h in presence of antibiotin microbeads and biotin CD3 and CD28 antibodies. Amounts of IFN-γ produced by CD8^+^ TCs were determined through enzyme-linked immunosorbent assay. (E) Effects of IL-19 (50 ng/mL) treatment on IFN-γ expression in CD4^+^ and CD8^+^ TCs stimulated with plate-bound CD3 antibody and soluble CD28 antibody for 48 h.


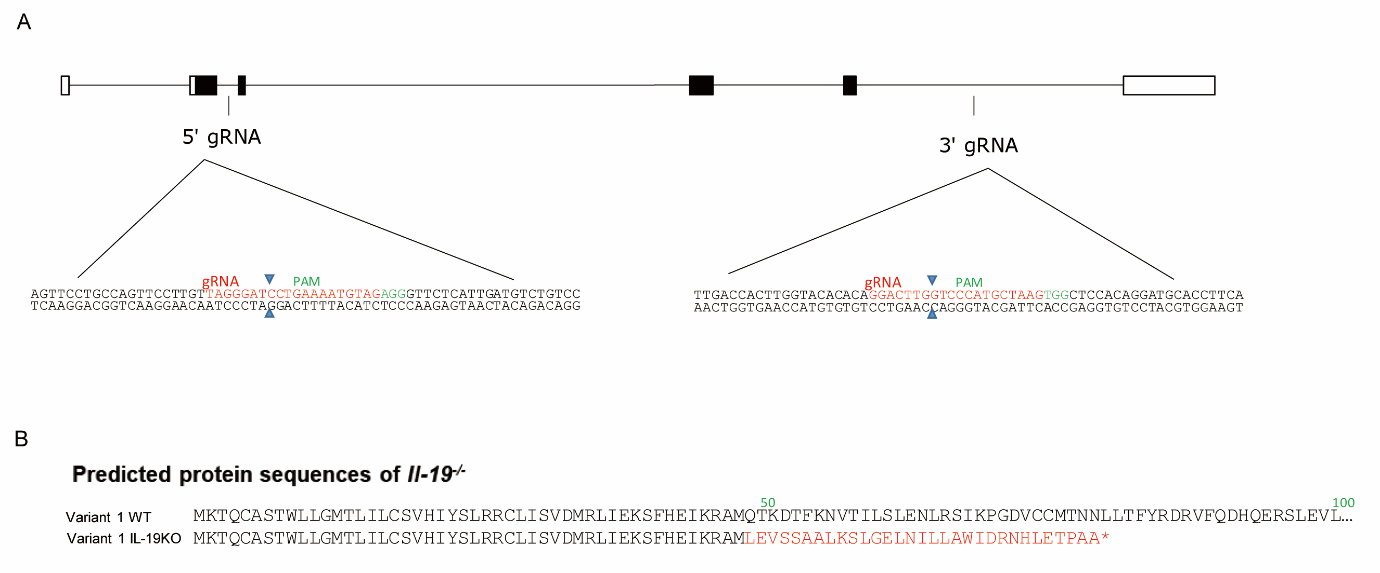


**Supplementary Fig.7 Generation of CRISPR *Il-19*^-/-^ knockout (IL-19KO) mice.**

(A) IL-19 knockout CRISPR design. The design of gRNA targeting in *Il-19* gene. The effect of gRNA targeting deletes the sequence between exon 2 and exon 4. (B) The predicted WT and IL-19KO protein sequences after CRISPR editing.


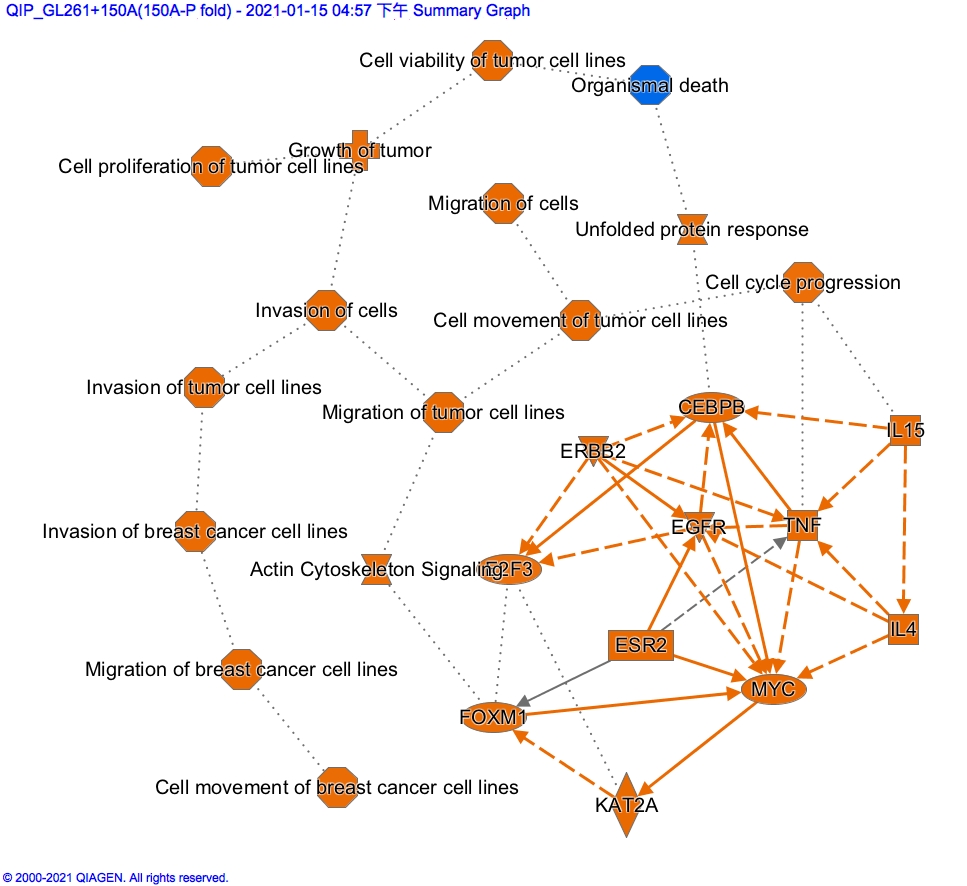


**Supplementary Fig. 8 Pathway analysis of the TMZ-resistant cell line.**

Cell lysates of GL261 and GL261/TMZ-R were subjected to proteomics analysis, revealing that GL261/TMZ-R cells had greater cell invasion, migration, and proliferation than TMZ-sensitive GL261 cells. The predicted pathways were analyzed by IPA. Orange color indicates the protein, signaling pathway, or function are predicted to be activation. Blue color indicates inhibition.

**
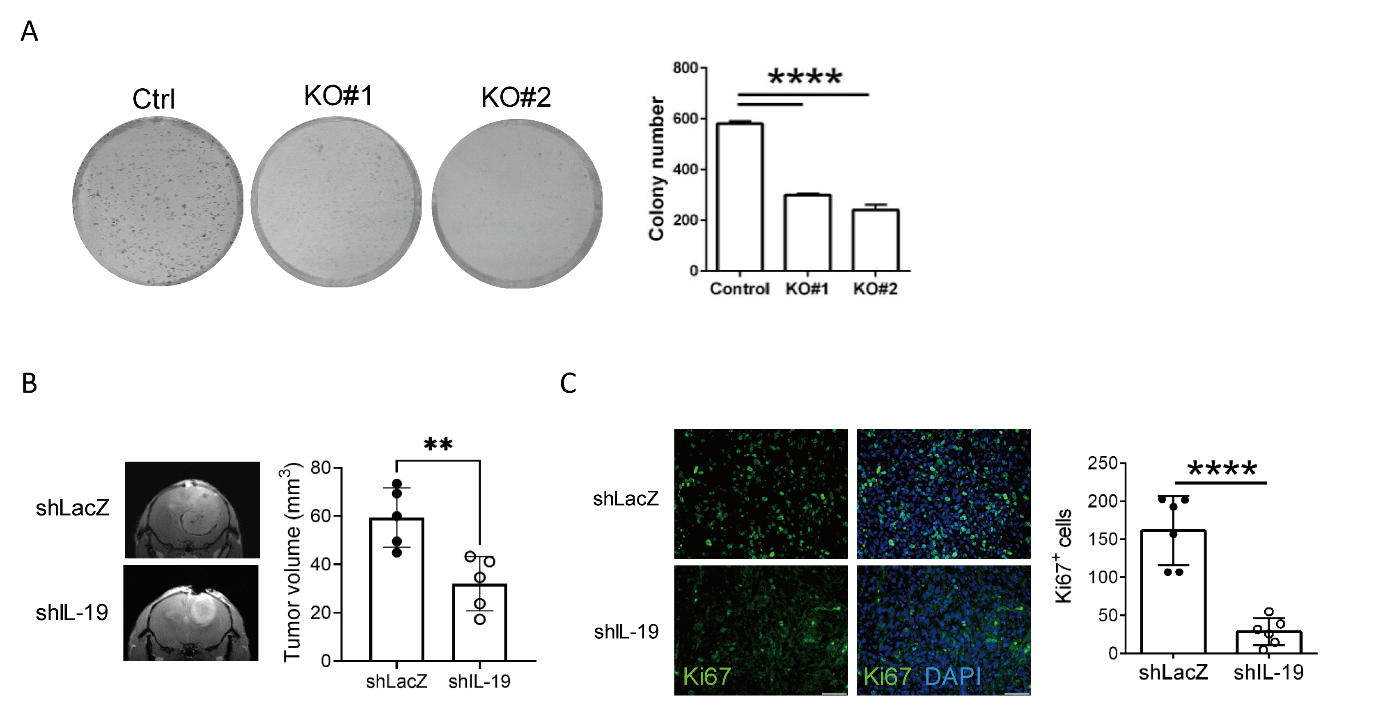
**

**Supplementary Fig. 9 Silencing IL-19 effects on GBM cell proliferation.** (A) Effects of silencing IL-19 on TMZ-resistant GBM colony formation ability. Representative images of control (Ctrl) and IL-19KO GL261/TMZ-R cell (KO#1 and KO#2) colonies are shown. Quantitative results of clonogenic assay expressed as total number of surviving colonies/well; n = 3, one-way ANOVA; ****p < .0001. (B) Representative post-contrast T1 MRI images of xenografts derived from shLacZ or shIL-19 GSCs. Tumor volumes were quantified at 13 days post-GSC inoculation. n=5 in each group. **p < .01 (C) Expression of the proliferation marker Ki67 in tumors derived from shLacZ and shIL-19 GSCs. Quantitative analysis of Ki67^+^ cell numbers in shLacZ- and shIL-19-derived tumors ****p < 0.0001.

**Supplementary Tables.**

**Supplementary Table 1. The detail of metagene lists for different immune subsets**

**
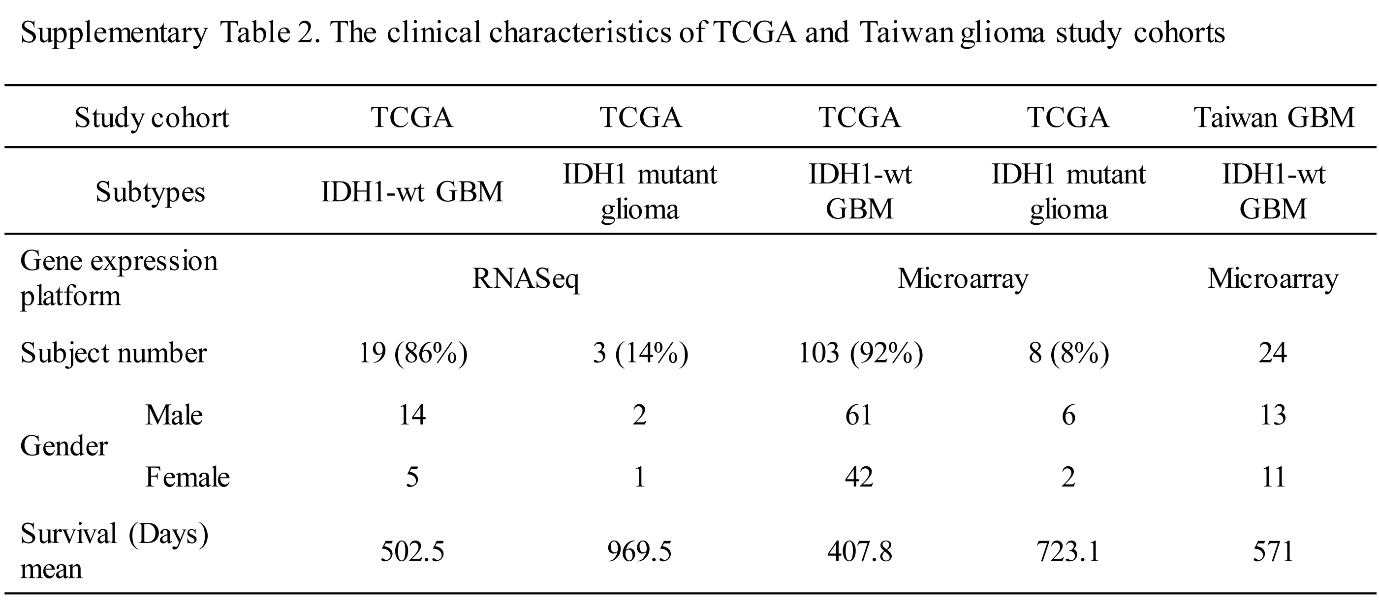
**

**Supplementary Table 2. Clinical characteristics of the TCGA and Taiwan GBM study cohorts.**
